# Gut Microbiota-Derived Tyrosol Alleviates Radiation-Induced Intestinal Injury via Targeting SCD1-MUFA Axis to Suppress ER Stress

**DOI:** 10.7150/ijbs.126269

**Published:** 2026-02-04

**Authors:** Xiaoya Jin, Hetian Xue, Xiaolin Shi, Yuchuan Zhou, Jialing Zhang, Liang Zeng, Xinglong Liu, Yuqi Xiao, Han Wang, Yue Zheng, Lina Wang, Yang Bai, Yan Pan, Jianghong Zhang, Yanwu Xu, Chunlin Shao

**Affiliations:** 1 Institute of Radiation Medicine, Shanghai Medical College, Fudan University, Shanghai, 200032, China.; 2 State Key Laboratory of Oncology in South China, Guangdong Provincial Clinical Research Center for Cancer, Sun Yat-sen University Cancer Center, Guangzhou, 510050, China.; 3 Radiotherapy Physics and Technology Center, Cancer Center, West China Hospital, Sichuan University, Chengdu, 610041, China.; 4 Department of Biochemistry, School of Integrative Medicine, Shanghai University of Traditional Chinese Medicine, Shanghai, 201203, China.; 5 Department of Radiation Oncology, Shanghai Proton and Heavy Ion Center, Fudan University Cancer Hospital, Shanghai, 201321, China.

**Keywords:** gut-derived metabolite, tyrosol, radioprotection, SCD1, endoplasmic reticulum stress

## Abstract

Radiation-induced intestinal injury (RIII) represents a major, clinically recalcitrant complication of radiotherapy, with current protective options remaining extremely limited. In this study, we identify tyrosol, a gut-derived phenolic metabolite enriched in the feces of irradiated mice, as a potent radioprotective agent. It reduced intestinal epithelial cell death and improved survival in lethally irradiated mice by preserving mucosal barrier and villus-crypt architecture, and downregulating pro-inflammatory cytokines. Mechanistically, we for the first time reveal that tyrosol directly targets stearoyl-CoA desaturase 1 (SCD1), a key enzyme involved in monounsaturated fatty acid (MUFA) biosynthesis. Tyrosol binds to conserved residues (Asn148, Asp156, Asn265) on SCD1, preventing valosin-containing protein (VCP)-mediated proteasomal degradation. This boosts SCD1 activity, increasing MUFAs (e.g., oleic acid, palmitoleic acid) to inhibit ER stress via the p-eIF2α/ATF4/CHOP axis and mitigate radiation-induced cytotoxicity. Importantly, inhibition of SCD1 in animal experiments abolishes tyrosol's protective effects, underscoring the essential role of SCD1. Additionally, MUFA supplementation rescues tyrosol's radioprotection in SCD1-deficient cells. These findings elucidate a novel mechanism whereby gut metabolites confer radioprotection through lipid remodeling and highlight SCD1 activation as a promising therapeutic strategy against gastrointestinal radiation injury.

## Introduction

The growing reliance on nuclear energy and radiation-based therapeutic modalities has heightened the urgency to develop innovative radioprotective strategies. Radiation-induced injury can stem from diverse sources, including radiological terrorism 1, nuclear disasters 2, occupational exposure 3, and cancer radiotherapy 4; all pose complex challenges to public health. Current clinical interventions, such as amifostine 5 and filgrastim 6, 7, modest protection against normal tissue damage but are often accompanied by substantial side effects and poor tolerability 8, 9, underscoring an urgent need for safer and more effective alternatives. The small intestine represents a primary target of radiation toxicity, with unique vulnerabilities: its rapidly proliferating epithelial mucosa exhibits highly sensitive to ionizing radiation, which can induce intestinal villus atrophy and mucosal barrier collapse. Moreover, barrier disruption enables translocation of intestinal bacteria and toxins into the systemic circulation, frequently culminating in life-threatening complications 10, 11. Despite decades of extensive research, progress in developing preventive and therapeutic approaches for intestinal radiation syndrome remains limited. This gap further emphasizes the critical need for natural or endogenous compounds that offer improved safety profiles and mechanism-driven targeted protection.

The gut microbiome and its metabolites play a pivotal role in regulating intestinal homeostasis and orchestrating responses to stress 12, 13. Recent studies have further established gut-derived metabolites as critical mediators that modulate both radiation-induced toxicity and tissue repair processes 14, 15. For example, short-chain fatty acids (SCFAs), products of microbial metabolism, regulate epithelial cell renewal and mitigate inflammation responses 11, 16. Tyrosol, a phenolic metabolite derived from the microbial metabolism of tyrosine by Escherichia species, has gained attention as a promising modulator of intestinal health 17. It enters the gastrointestinal tract through two primary pathways: exogenous dietary intake and endogenous microbial metabolism. As a major phenolic compound found in extra virgin olive oil, dietary tyrosol exists in both free and esterified forms, the latter conjugated with long- or short-chain fatty acids. Free tyrosol is rapidly absorbed in the small intestine, reaching peak concentrations, while esterified tyrosol remains stable within the gastric environment and is hydrolyzed by intestinal lipases in the small intestine to release free tyrosol, facilitating a sustained-release effect 17, 18. These synergistic pathways—dietary absorption and microbial metabolism—collectively govern the *in vivo* levels and metabolic fate of tyrosol, underscoring its potential significance in maintaining intestinal health. Preclinical studies have consistently linked tyrosol to potent anti-inflammatory effects 19, 20, as well as protective roles against gastrointestinal (GI) disorders including ulcerative colitis 21, 22 and colorectal cancer 23. Notably, tyrosol is endogenously produced by the gut microbiota 23 and has a low toxicity profile, rendering it a particularly attractive candidate for developing radioprotective strategies.

Recent advances have emphasized the pivotal role of metabolic reprogramming in mediating cellular stress responses, with particular focus on the regulation of lipid metabolism 24-26**,** endoplasmic reticulum (ER) stress 27, 28, and protein homeostasis 29. Particularly, excessive ER stress induced by irradiation has been linked to poor clinical outcomes, highlighting the need to identify regulators that can mitigate such stress-related damage.

Stearoyl-CoA desaturase 1 (SCD1), a key ER-anchored lipid desaturase, serves as a central hub in coordinating lipid metabolism, ER stress homeostasis, and intestinal epithelial integrity—making it a critical mediator in intestinal injury responses. As the rate-limiting enzyme that catalyzes the conversion of saturated fatty acids (SFAs) to monounsaturated fatty acids (MUFAs) such as oleic acid and palmitoleic acid 30, 31, SCD1 exerts direct and indirect regulatory effects on ER function, with profound implications for stress resistance and intestinal health. Mechanistically, SCD1 activity is tightly intertwined with ER stress signaling: it not only modulates the lipid composition of ER membranes but also engages in a reciprocal regulatory loop with the unfolded protein response (UPR) 32, 33. In the context of intestinal injury, SCD1 dysfunction exacerbates ER stress and mucosal damage: its inhibition leads to SFA accumulation in ER membranes, disrupting membrane fluidity and integrity 34, 35, which further amplifies ER stress. The protective effects of SCD1 are largely mediated by its downstream product, MUFAs. MUFAs not only stabilize ER membrane structure by balancing the SFA/MUFA ratio 36, 37 but also directly attenuate ER stress signaling cascades—counteracting the pro-stress effects of SFAs 31, 38. In the intestine, MUFAs derived from SCD1 activity have been shown to reinforce epithelial tight junctions, reduce pro-inflammatory cytokine production, and mitigate mucosal barrier disruption, whereas dysregulated SCD1-MUFA axis disrupts ER function and exacerbates intestinal damage in pathological models 39, 40. Beyond ER stress regulation, SCD1 activity is implicated in a range of pathological processes, including metabolic disorders 41 and cancer progression 31, underscoring its pleiotropic role in cell adaptation to environmental perturbations. Collectively, these findings establish SCD1 as a critical modulator of intestinal ER stress and mucosal integrity, with its protective functions largely dependent on MUFA synthesis. But the specific role of this SCD1-MUFA-ER stress axis in radiation-induced intestinal injury (RIII) remains uncharted.

In this study, we employed untargeted metabolomics to investigate gut metabolic alterations following irradiation, leading to the identification of tyrosol, an upregulated metabolite, as a potential mediator of intestinal defense. We further explored the mechanistic role of tyrosol in ameliorating RIII, focusing on its interaction with SCD1, influences of protein degradation, facilitates monounsaturated fatty acids (MUFAs) synthesis and modulation of ER stress. Our findings revealed a novel protective mechanism against radiation, centered on tyrosol-mediated stabilization and activation of SCD1. Tyrosol interfered with valosin containing protein (VCP)-dependent proteasomal degradation of SCD1, thereby facilitating MUFA synthesis to attenuate ER stress and promote epithelial cell survival. These insights advance our understanding of how targeting lipid metabolism and stress response pathways can mitigate radiation-induced GI injury.

## Materials and Methods

### Animal procedures, irradiation, and drug administration

Male C57BL/6J mice (8-10 weeks old, ~22 g) were obtained from SPF Biotechnology co., LTD (Suzhou, China). Animals were housed under a 12-h light/dark cycle at 22-24 °C with 40-60% humidity, with free access to food and water. The procedures were sanctioned by the Animal Care and Use Committee of Shanghai Medical College, Fudan University.

Mice were irradiated with X-rays using an X-RAD 320 system (Precision X-ray, Inc., North Branford, CT, USA) at 2 Gy/min, with a source-to-skin distance of 60 cm. The beam (320 keV, 12 mA) was filtered with 2.5 mm aluminum and 0.1 mm copper. Total body irradiation (TBI) was delivered as a 7 Gy single dose, while total abdominal irradiation (TAI) was administered as 12 Gy, with the irradiation restricted to the area between the xiphoid and pubic symphysis and the remaining body parts shielded by a 15-mm lead plate. The 12 Gy TAI dose was selected based on biological effective dose (BED) calculations using the Linear-Quadratic model (α/β = 8 Gy 42) to approximate the cumulative mucosal injury associated with clinical regimens of 50 Gy delivered in 2 Gy fractions. This single-dose protocol is well-established, as doses between 10 and 15 Gy reliably induce acute intestinal injury, such as crypt stem cell loss and villous atrophy, within 48-72 h 10, 43-45, thereby modeling early radiation-induced damage and providing a suitable platform for studying epithelial repair and interventions 14, 46. Post-irradiation, mice were monitored for survival and body weight over 20 days.

For pharmacological interventions, tyrosol (188255, Sigma) was dissolved in saline and administered orally at 20, 50 or 75 mg/kg daily in a volume of 75-100 μL (not exceeding 5 mL/kg). The SCD1 inhibitor A939572 (T4515, TargetMol) was dissolved in dimethyl sulfoxide as a 150 mg/mL stock. The mice were administered A939572 daily by oral gavage (30 mg/kg diluted in corn oil) at 3 days before tyrosol treatment. The L-Tyrosine (T8566, Sigma) was dissolved in drinking water at concentrations of 100 mg/kg or 500 mg/kg, starting 10 days prior to total abdominal irradiation (TAI, 12 Gy) and continuing until 4 days post-irradiation.

### Untargeted metabolomics

Fecal samples were collected under sterile conditions, rapidly frozen in liquid nitrogen, and stored at -80 °C. Samples (~60 mg) were homogenized with steel beads in 600 μL of a methanol-water solution (4:1, v/v) containing 4 μg/mL L-2-chlorophenylalanine as an internal standard. The extraction process included cryogenic homogenization (60 Hz, 2 min), ultrasonication (ice bath, 10 min), and incubation at -40 °C for protein precipitation. After centrifugation (15500 g, 10 min, 4 °C), supernatants were lyophilized, reconstituted in 300 μL methanol-water (1:4, v/v), vortexed, sonicated (ice bath, 3 min), and filtered through 0.22 μm membranes.

Metabolomic profiling utilized an ACQUITY UPLC I-Class system (Waters Corporation, Milford, USA) coupled with a VION IMS QTOF mass spectrometer. Separation was on a BEH C18 column (1.7 μm, 2.1×100 mm) with a water and acetonitrile/methanol gradient, both containing 0.1% formic acid. Analyses utilized positive and negative electrospray ionization in MSE mode, with an injection volume of 1 μL at 4 °C. Source parameters included a capillary voltage of 2.5 kV, source temperature of 115 °C, and desolvation gas flow at 900 L/h (450 °C).

Data processing was conducted using Progenesis QI v2.3 against standard metabolite databases (HMDB, Lipid Maps, Metlin). Features with > 50% missing data were excluded; zero values were imputed as half the minimum detected intensity. Differential metabolites (VIP > 1.0, p < 0.05) were identified via PCA, OPLS-DA or PLS-DA, validated through cross-validation and permutation testing.

### Absolute quantification of tyrosol

HPLC analysis of tyrosol in feces, intestinal contents, and small intestinal tissue was conducted as previously described. Briefly, samples (100 mg feces or intestinal content, 200 mg small intestinal tissue) were homogenized in 0.5 mL of anhydrous ethanol, sonicated thoroughly, then dried under nitrogen gas. The residues were reconstituted in sterile water, centrifuged at 23,000 g at 4 °C for 1 h, and the supernatant filtered through a 0.22 μm membrane prior to analysis.

HPLC was performed using a Dionex P680 quaternary pump (Sunnyvale, USA), Gynkotek Gina 50 autosampler (5 °C), Gynkotek UVD 340S diode array detector (Gynkotek, Macclesfield, Cheshire), and Chromeleon software. An injection volume of 100 μL was used, with a flow rate of 1.0 mL/min. Eluent absorbance was monitored at 280 nm, and tyrosol concentrations were quantified via linear regression analysis.

### 16S rRNA amplicon sequencing

Genomic DNA was extracted from samples using the MagPure Soil DNA LQ Kit (Magan). DNA was stored at -20 °C. The V3-V4 regions of the 16S rRNA gene were amplified using barcoded primers 343F and 798R, with additional amplification using primers 907R for region coverage, and Takara Ex Taq polymerase (Takara). PCR products were gel-purified with AMPure XP beads (Agencourt), quantified with Qubit, and pooled for sequencing. Paired-end sequencing (250 bp) was performed on an Illumina NovaSeq 6000 platform (Illumina Inc., San Diego, CA; OE Biotech Company; Shanghai, China). Sequencing data were processed by OE biotech Co., Ltd. (Shanghai, China). Raw FASTQ files were adapter-trimmed with Cutadapt, then filtered, denoised, and merged using DADA2 within QIIME 2 (2020.11). Chimeras were removed, generating representative ASVs and abundance tables. Taxonomic annotation was performed using QIIME2 with the Silva database.

### Blood routine tests

On Day 7 after TBI, anticoagulant blood was taken for routine blood test provided by Servicebio company.

### Inflammatory factors detection in mouse serum

To quantify IL-10, IL-1β, TNF-α, and IL-6 in mouse serum, the XMplex Mouse 4-Plex Custom Panel (#XMPlex01240648, XM-BIOTECH) and XMplex-100 analyzer were used. Add 5 μL microsphere suspension and 50 μL standards or serum samples to each well, incubate at 37°C for 1 h, and wash once. Then add 50 μL detection antibody, incubate at 37 °C for 30 min, and wash. Add 50 μL fluorochrome solution, incubate at 37 °C in the dark for 15 min, add 55 μL wash buffer, and detect with XMplex-100. Target concentrations were calculated via standard curves (linear range: 6.86-5000 pg/mL).

### Cell culture, irradiation, and drug treatment

Human intestinal epithelial cell (HIEC), intestinal epithelioid cell (IEC-6), and human embryonic kidney 293T (HEK-293T) cells were acquired from the Cell Bank of Chinese Academy of Sciences (Shanghai, China) and cultured in DMEM (Gibco, CA, USA) supplemented with 10% FBS (Gibco, Thermo Fisher Scientific, Waltham, MA, USA), penicillin (100 U/mL, Gibco), streptomycin (100 μg/mL, Gibco), and insulin (TargetMol, 10 μg/mL for IEC-6). Cells were cultured at 37 °C in 5% CO₂. Mycoplasma testing and short tandem repeat profiling was performed to confirm cell authenticity prior to experiments. Cells in logarithmic growth phase were irradiated with X-rays at 1 Gy/min.

For drug treatment, tyrosol was added in cell culture medium 24 h prior to irradiation. Cells were treated with 4-phenylbutyric acid (4-PBA; T5886, TargetMol, ER stress inhibitor, 1mM), Tauroursodeoxycholate dihydrate (TUDCA; T16998, TargetMol, ER stress inhibitor, 50 μM), Thapsigargin (TG; TQ0302, TargetMol, ER stress inducer, 10nM), Liproxstatin-1 (HY-12726, MCE, ferroptosis inhibitor, 0.2 μM), Necrostatin-1 (HY-15760, MCE, necroptosis inhibitor, 1 μM), PluriSln #1 (S8076, Selleck, SCD1 inhibitor, 20 μM), A939572 (T4515, TargetMol, SCD1 inhibitor, 20 μM), or VCP inhibitor NMS-873 (T1853, TargetMol, 1-10 μM) 2 h before irradiation. For fatty acid treatment, cells were incubated with 75 μM of individual fatty acids—oleic acid (KC005), stearic acid (KC007), or palmitoleic acid (KC010, all from Kunchuang Biotechnology)—or an equal volume of solvent control for 24 h before exposure to irradiation. To assess protein stability, cells were treated with Cycloheximide (CHX; T1225, TargetMol, protein synthesis inhibitor, 10 μM), MG132 (HY-13259, MCE, proteasome inhibitor, 10 μM), Leupeptin (HY-18234, MCE, lysosomal protease inhibitor, 10 μM), or TAK-243 (T16974, TargetMol, ubiquitin-activating enzyme inhibitor, 10 μM) 2 h prior to irradiation or cell collection.

### Cell viability assay

Cell viability was assessed via the Cell Counting Kit-8 (CCK8) assay (CK04, Dojindo). Cells were seeded in 96-well plates at a density of 8,000 cells per well. After overnight culture, different treatments were applied. At the end of the experiment, the medium was replaced with fresh medium containing CCK-8 and incubated at 37 °C for 2 h. The absorbance was measured at 450 nm using a Tecan Infinite M200 Pro reader.

### Lactate dehydrogenase (LDH) release assay

Cytotoxicity was evaluated with the LDH assay (C0017, Beyotime,). Cells in 24-well plates (3-5×10⁴ cells/well) were treated for 2 h or 24 h. Supernatants were collected post-centrifugation (400×g, 5 min). The LDH activity test was carried out according to the instructions of the kit. Absorbance was read at 490 nm with background correction at 630 nm.

### Calcein-AM/PI staining

Cell viability and death were visualized using a Calcein-AM/PI kit (C326, Dojindo). At the experiment endpoint, cells (1×10⁵/well in 24-well plates) were stained with 2 μM Calcein-AM and 4.5 μM PI in PBS at 37°C for 30 min. Fluorescence imaging was performed by fluorescence microscope to quantify live (green) and dead (red) cells, with four fields per well analyzed.

### Histology

Following euthanasia, the mice's small intestines, liver, and kidney were fixed in 4% buffered formalin at room temperature overnight before being embedded in paraffin. Sections (5 μm) were prepared and stained with hematoxylin and eosin (H&E) and Periodic acid-Schiff (PAS) following standard protocols.

### Biochemical analysis of liver and kidney function markers

Mice blood was collected via cardiac puncture under sterile conditions and allowed to clot at room temperature for 30-60 min before centrifugation at 1000 g for 10 min to isolate serum. Biochemical parameters such as alanine aminotransferase (ALT), aspartate aminotransferase (AST), total bilirubin (TBIL), blood urea nitrogen (BUN), creatinine (CREA), and uric acid (UA) were measured using commercial assay kits (GM1102, GM1103, GM1105, GM1110, GM1112, GM1111, Servicebio). Reactions were conducted according to manufacturer protocols, with NADH consumption or chromogenic product formation measured at specific wavelengths at 37 °C.

### RNA extraction and qRT-PCR

The extraction of total RNA from cells was performed using Trizol reagent (Invitrogen), followed by reverse transcription into cDNA with the FastKing RT Kit (Tiangen Biotechnology). Using SuperReal PreMix Plus (Tiangen Biotechnology), the qRT-PCR was executed on the Bio-Rad CFX Opus 96 platform. ACTB served as the endogenous control. Primer sequences are provided in Supplementary Table S1.

### GSH measurement

The content of glutathione (GSH) was determined by GSH and GSSG Detection Kit (S0053, Beyotime). The level of GSH in HIEC cells was detected using a GSH and Glutathione Disulfide (GSSG) Assay kit following the manufacturer's instructions.

### Endoplasmic reticulum stress reporter gene assay

HIEC cells were transduced with REPO^TM^ERSE lentiviral reporter constructs (GM-022047, Genomeditech) and selected with puromycin to establish stable expression. Following a 24-h pre-treatment with tyrosol, cells were exposed to 8 Gy irradiation. Reporter activity was measured using the firefly luciferase assay kit (RG005, Beyotime), performed in accordance with the manufacturer's protocol.

### Intestinal permeability assay

On day 3 post-irradiation, mice were fasted for 4 h with ad libitum water. FITC-dextran (46944, Sigma) was administered orally at 50 mg/100 g body weight. Blood samples were collected at 2 h post-gavage, and serum fluorescence was measured using a microplate reader (excitation at 490 nm; emission at 520 nm). Fluorescence intensity was normalized to the mean value of the non-irradiated control group.

### Prediction and analysis of potential targets for tyrosol's protective effect

A network pharmacology strategy was employed to predict and gather potential targets of tyrosol and disease-related targets. The molecular structure of tyrosol was obtained from PubChem in Canonical SMILES or sdf format and subsequently submitted to publicly available target prediction platforms, including ChemMapper, HERB, SEA, SwissTargetPrediction, and SuperPred, to infer its putative targets. Targets associated with endoplasmic reticulum (ER) stress were retrieved from GeneCards and the ALLIANCE database. The intersection of tyrosol-related and ER stress-related targets was considered as the candidate target set potentially linked to its protective effects. To further prioritize these candidates, DeepLoc-2.1 was used to predict their subcellular localization, particularly ER enrichment scores, and the results were visualized using GraphPad Prism 9.0. Finally, by integrating their expression profiles in the small intestine, key target proteins potentially mediating tyrosol's protective effects were identified. The specific versions and access details of the web-based tools used are provided in Supplementary Table S2.

### Lentiviral transduction and siRNA transfection

The wild-type SCD1 and its mutants (N148G, D156G, N265G) were cloned into the pLV-CAG-Puro/BSD vector, with an N-terminal Flag tag. shRNAs targeting SCD1, with the sequence CTACGGCTCTTTCTGATCATT (shSCD1-1, selected for subsequent experiments), CCCACCTACAAGGATAAGGAA (shSCD1-2), CGTCCTTATGACAAG AACATT (shSCD1-3) were inserted into the pLKO.1 vector. Lentiviruses were then generated by co-transfecting these constructs or vectors along with psPAX2 and pMD2.G into HEK293T cells. Subsequently, HIECs were infected with these lentiviruses and subjected to puromycin (ST551, Beyotime) or Blasticidin S HCl (ST018, Beyotime) selection to establish stable cell lines. For VCP knockdown, HIECs were transfected with siRNA or non-targeting controls using Lipofectamine RNAi MAX (13778150, Thermo Fisher Scientific), with target sequence CTTATCTCACCACGAATTC, and analyzed after 72 h.

### Western blotting

Cells were lysed using RIPA buffer (P0013B, Beyotime). Equal protein amounts were resolved by SDS-PAGE and transferred onto PVDF membranes (ISEQ00010, Merck). After blocking with 5% nonfat milk in TBST (G2150-1L, Servicebio), membranes were incubated with primary antibodies for 2 h at room temperature. Following washes, membranes were probed with HRP-conjugated secondary antibodies (A0208, A0216, Beyotime; 1:5000) and visualized using an ECL detection system. The list of primary antibodies is provided in Supplementary Table S3.

### Molecular docking and molecular dynamics (MD) simulations

Molecular docking was employed to assess the binding affinity of tyrosol to SCD1. First, the SDF format structure of tyrosol was converted to PDB format using Open Babel 2.3.2. The SCD1 structure (PDB ID: 4ZYO) was downloaded from RCSB PDB. Ligand and solvent molecules were removed using PyMOL 2.3.4. Hydrogenation was performed with AutoDock Tools 1.5.6. Then, the ligand and receptor files were converted into pdbqt format, and docking was carried out with AutoDock Vina 1.1.2. The optimal docking mode was selected and visualized using either PyMOL (v3.17) or LigPlot+ (v2.2.9).

Molecular dynamics (MD) simulations were performed with the GROMACS 2023.2 software in single precision mode. Small molecules were pre-processed with the Amber99SB force field. Subsequently, the data were processed into a topological file to prepare for MD simulations. The MD simulations were conducted at a temperature of 300 K and a pressure of 1 bar. Using the TIP3P model, water molecules acted as the solvent, and Na⁺ ions were added to achieve a neutral total charge for the system. Energy minimization was achieved through the steepest descent approach, succeeded by 100,000 steps of isothermal-isovolumetric and isothermal-isobaric equilibration. A free MD simulation of 5,000,000 steps (with a step size of 2 fs) was conducted, resulting in a total simulation time of 100 ns. The results were then analyzed and visualized using Qtgrace 2.6. The analysis included the calculation of root-mean-square deviation (RMSD), radius of gyration, hydrogen bonds frequency and the Δ molecular mechanics-generalized-Born surface area (ΔMMGBSA).

### Drug affinity responsive target stability (DARTS) assay

DARTS was conducted following the previously outlined method 47. Cells were lysed with NP-40 buffer, and protein concentrations were quantified using the BCA Protein Assay Kit (P0009, Beyotime). Lysates were incubated with 3 μL tyrosol (final concentration 2 mM) or vehicle at 4 °C for 2 h. Samples were then treated with 1 μg/mL Pronase (Roche) or distilled water for specified durations. Following proteolysis, proteins were mixed with 5× loading buffer (P0015L, Beyotime), boiled at 95 °C for 10 min, and subjected to western blot analysis.

### Analysis on the cross-species conservation of receptor-ligand binding sites

Retrieve and download the FASTA files of the SCD1 protein sequences for human, monkey, rat, and mouse from NCBI. Submit them to Clustal Omega for sequence comparison and visualization. Then, evaluate the cross - species conservation of key amino acid residues at the binding sites between tyrosol and SCD1 based on molecular docking results.

### Fatty acid content determination

Cell samples were transferred to 2 mL tubes with 1 mL chloroform-methanol (2:1) and glass beads, homogenized at 55 Hz, and centrifuged (15500 g, 10 min, 4 °C). Supernatants were treated with 1% sulfuric acid-methanol for esterification (80 °C, 30 min), extracted with n-hexane. After centrifugation (1200 g, 10 min, 4 °C), supernatants were dried with sodium sulfate, spiked with methyl salicylate (internal standard), and analyzed by GC-MS. GC-MS used a TG-FAME column (50 m × 0.25 mm) with helium at 0.63 mL/min. The temperature program ranged from 80-250 °C with specified ramps. Detection employed electron impact ionization (70 eV) in SIM mode.

### Co-immunoprecipitation (Co-IP)

Whole-cell lysates were prepared and centrifuged at 10,000 × g for 10 min at 4 °C. Clarified lysates (1 ml) were incubated overnight with specific primary antibodies, followed by the addition of 20 μl protein A/G agarose beads (sc-2003, Santa Cruz Biotechnology, Inc.) and incubation at 4 °C for an additional 16 h. Beads were washed four times with immunoprecipitation buffer, and proteins were eluted for analysis by SDS-PAGE and western blotting. Antibodies used are listed in Supplementary Table S3**.**

### Immunofluorescence (IF) staining

Cells underwent fixation with 4% formaldehyde for 10 min, then permeabilized with 0.5% Triton X-100 (ST1723, Beyotime) for another 10 min, and subsequently blocked for 1 h in PBS containing 0.1% Tween-20, 1% BSA, 10% normal goat serum, and 0.3 M glycine (QuickBlock, P0260, Beyotime). Overnight incubation of cells at 4°C was done with primary antibodies, followed by a one-hour incubation with secondary antibodies. The nuclei of the cells were stained with DAPI (C1006, Beyotime).

### Statistical analysis

Data are expressed as mean ± standard deviation. Survival analyses utilized Kaplan-Meier curves generated with GraphPad Prism 9.0. Differences between two groups were assessed by Student's t-test, while multiple comparisons employed one-way ANOVA with Tukey's post hoc test. A *p*-value < 0.05 was considered statistically significant.

## Results

### Irradiation altered intestinal metabolite profile

**Figure 1:**
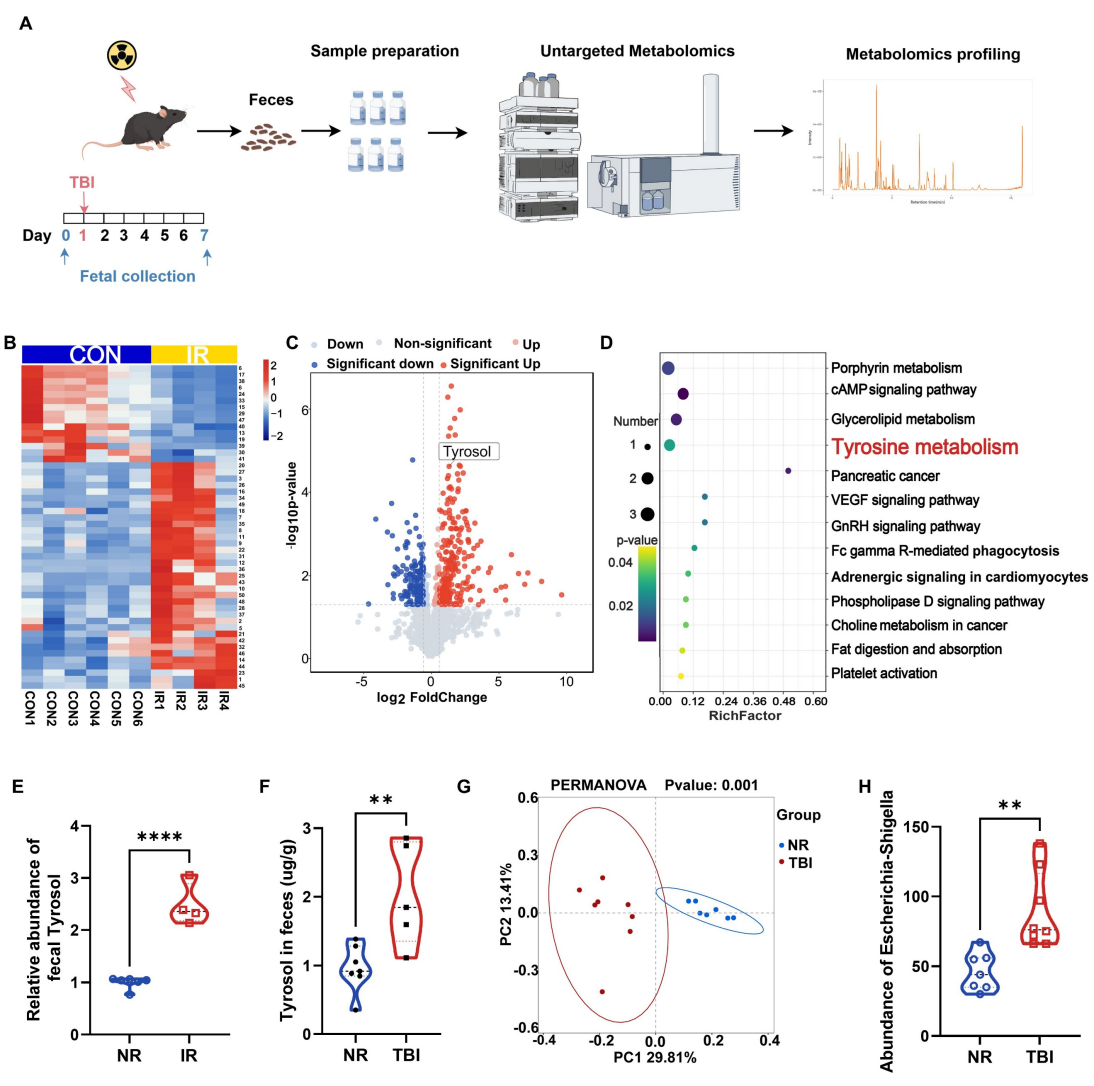
** Mice exposed to irradiation develop a distinct intestinal metabolite profile compared to non-irradiated controls.** (A) Schematic of fecal metabolite analysis of TBI-treated mice: SPF C57BL/6 mice received 7 Gy TBI, feces collected at day 0 (NR) and 7 (IR 7d). (B) Heatmap of differential metabolites of NR vs IR 7 d fecal samples. A detailed metabolites list is provided in Supplementary Table S4. (C) Volcano plot of significant metabolite changes of post-TBI feces (NR vs IR 7d). (D) GO enrichment analysis bubble plot of significantly enriched pathways among upregulated metabolites. (E) Relative abundance of tyrosol in feces. (F) Absolute quantification of tyrosol in mouse feces. (G) PCoA plot of microbial composition. (H) Abundance of Escherichia-Shigella in mice feces. Each point representing an individual mouse. Bars represent mean ± SD. TBI, total body irradiation. ****p < 0.0001.

To investigate irradiation-induced alterations in intestinal metabolites, we collected fecal samples from irradiated and non-irradiated mice for untargeted metabolomic analysis. A critical limitation was encountered: mice treated with a lethal dose of TAI displayed reduced intestinal contents and compromised defecation, severely hindering fecal sample collection. Conversely, lowering the total abdominal irradiation (TAI) dose failed to induce significant intestinal inflammatory infiltration or tissue damage. To resolve this constraint and optimize fecal sampling, we referenced previous studies 14, 46 and administered 7 Gy of total body irradiation (TBI) to mice, collecting fecal samples at two time points: 1 day before irradiation and 7 days after irradiation (Fig. 1A). The seventh day post-TBI was selected based on prior studies 48, 49, which identify it as a key period for microbiota alterations associated with host recovery. Untargeted metabolomics analysis of the samples identified 426 differentially expressed metabolites (fold change > 1.5, *P* < 0.05, VIP > 1), of which 232 were upregulated and 194 were downregulated (Fig. 1B, C). KEGG pathway analysis revealed significant perturbations in porphyrin metabolism, glycerolipid metabolism, tyrosine metabolism, and the cAMP signaling pathway (Fig. 1D). Notably, 4 out of 6 key metabolites in the porphyrin metabolism, glycerolipid metabolism, and cAMP signaling pathways lacked commercially available standards; the remaining two were metabolically unstable *in vivo*, undergoing rapid degradation to other substances and thus failing to maintain effective plasma concentrations. We therefore focused on tyrosine metabolism. Within this pathway, a notable change was observed: fecal level of tyrosol, an intermediate metabolite therein, was increased in the irradiated mice (Fig. 1E). And quantitative HPLC analysis further confirmed this change in fecal tyrosol content in TAI mice (Fig. 1F).

16S rRNA sequencing of fecal samples demonstrated profound alterations in the gut microbiota composition following TBI (Fig. 1G), with a marked enrichment of the tyrosol-producing bacterium *Escherichia-Shigella* (Fig. 1H). These findings suggest a potential adaptive role of tyrosol in radiation-induced intestinal injury (RIII). Importantly, tyrosol, a metabolite derived from tyrosine, has been extensively reported to possess anti-inflammatory activity 19, 20, along with protective roles against diarrhea, ulcerative colitis, and colorectal cancer 21, 23, 50, indicating its potential to mediate radioprotection.

### Tyrosol ameliorates TBI induced systemic injuries

**Figure 2:**
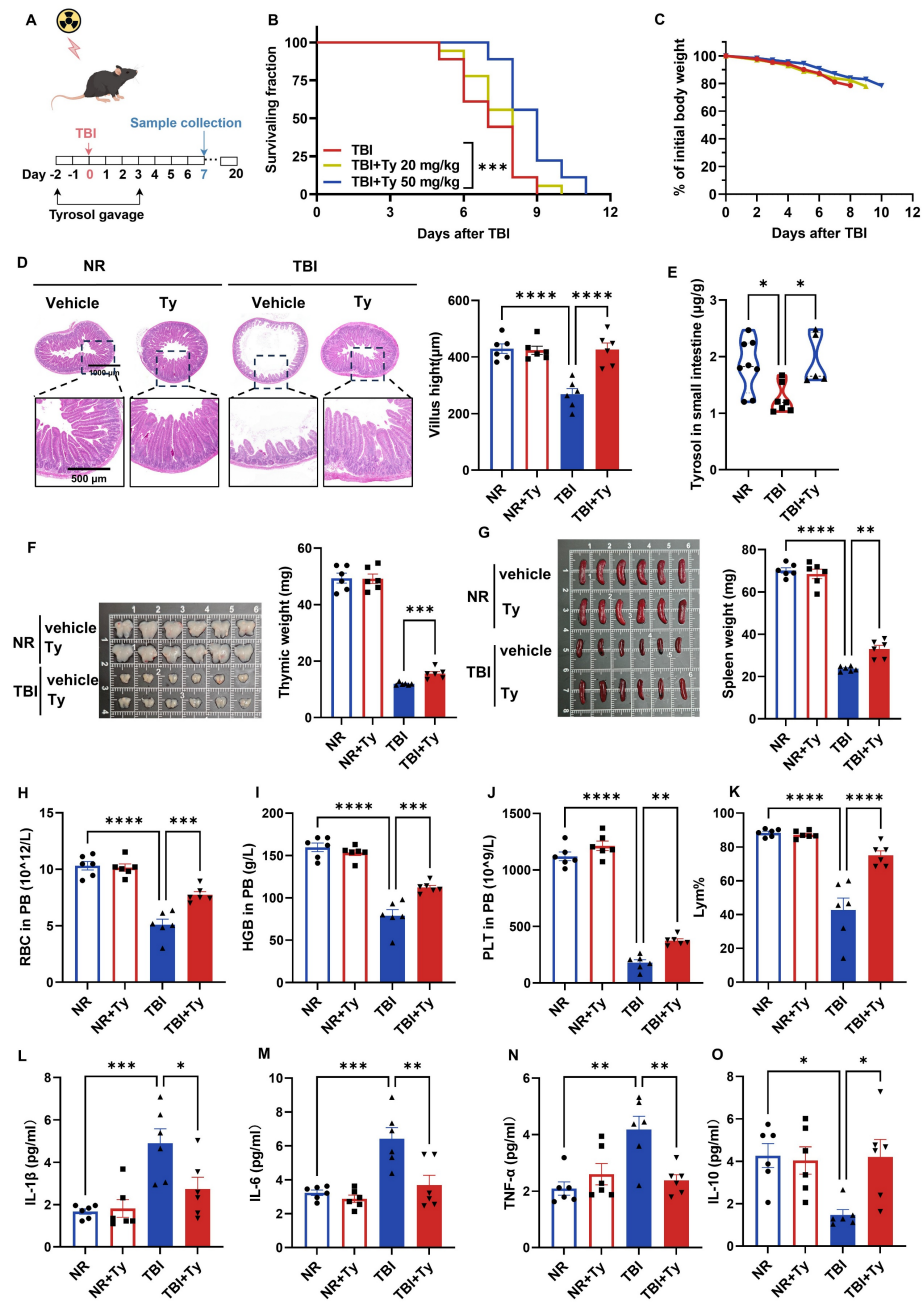
** Oral gavage of tyrosol ameliorates TBI induced systemic injuries.** (A) Schematic of tyrosol administration (20 or 50 mg/kg) via gavage to ameliorate radiation-induced injury. Mice were monitored over 20 days or euthanized at day 7 post-7 Gy TBI. (B-C) Survival curves (B) and body weight changes (C) of irradiated mice. (n = 18 per group). (D) Representative H&E-stained intestinal sections at day 0 and 7 post 7Gy TBI; right, quantification of villus height. (E) Absolute quantification of tyrosol in mouse small intestine. (F-G) Photographs of thymuses(F) and spleens (G) at day 0 and 7 post 7Gy TBI; right, quantification of thymuses or spleen weight. (H-K) Red blood cell (RBC) counts (H), haemoglobin (HGB) concentration (I), platelet (PLT) counts (J) and percentage of lymphocytes (Lym%) (K) in PB were measured at day 7 after 7 Gy TBI. (L-O) ELISA analysis of IL-1β(L), IL-6(M), TNF-α(N) and IL-10 (O) content in PB at day 7 post-TBI. Each symbol represents one mouse. Bars represent mean ± SD. Ty, tyrosol; TBI, total body irradiation; PB, peripheral blood. *p < 0.05, **p < 0.01, ***p < 0.001, ****p < 0.0001.

Because accidental irradiation often involves total-body exposure, we evaluated the radioprotective efficacy of exogenous tyrosol in a 7 Gy total-body irradiation (TBI) model. Mice received tyrosol by daily oral gavage from 2 days before irradiation to 3 days after irradiation (Fig. 2A). High-dose tyrosol (50 mg/kg) prolonged survival (Fig. 2B) and attenuated TBI-induced weight loss (Fig. 2C), demonstrating robust radioprotection.

On day 7 post-TBI, the small intestine, thymus, spleen, and peripheral blood (PB) were collected for further analyses. Tyrosol markedly mitigated TBI-induced villus atrophy (Fig. 2D) and restored intestinal tyrosol levels, which declined after irradiation but were replenished by exogenous administration (Fig. 2E), supporting protection against radiation-induced enteropathy. Tyrosol also attenuated thymic and splenic atrophy (Fig. 2F, G) and preserved hematological indices, reducing TBI-associated declines in red blood cells, hemoglobin, platelets, and lymphocyte proportion in PB (Fig. 2H-K). Consistently, tyrosol blunted systemic inflammation, decreasing serum IL-1β, IL-6, and TNF-α while increasing IL-10 (Fig. 2L-O). Together, these data demonstrate that tyrosol provides systemic radioprotection under TBI.

Meanwhile, we conducted a preliminary *in vivo* safety assessment of tyrosol. Briefly, mice received tyrosol by daily oral gavage at 20, 50, or 75 mg/kg for five consecutive days. Serum biochemical markers of hepatic and renal function, together with H&E-based histopathological evaluation of the liver and kidney, showed no apparent toxicological changes at any dose (Fig. S1A, B), indicating a favorable safety profile of tyrosol at the tested doses.

The bone marrow and intestine are among the most severely affected target organs after TBI. Although hematopoietic stem cell transplantation can effectively restore hematopoiesis, effective pharmacological or clinical interventions for radiation-induced intestinal injury (RIII) remain limited. Given that the intestine is the first tissue directly exposed to microbiota-derived metabolites, we next focused on the protective potential of tyrosol against gastrointestinal radiation injury.

### Tyrosol attenuates RIII *in vitro* and *in vivo*

**Figure 3:**
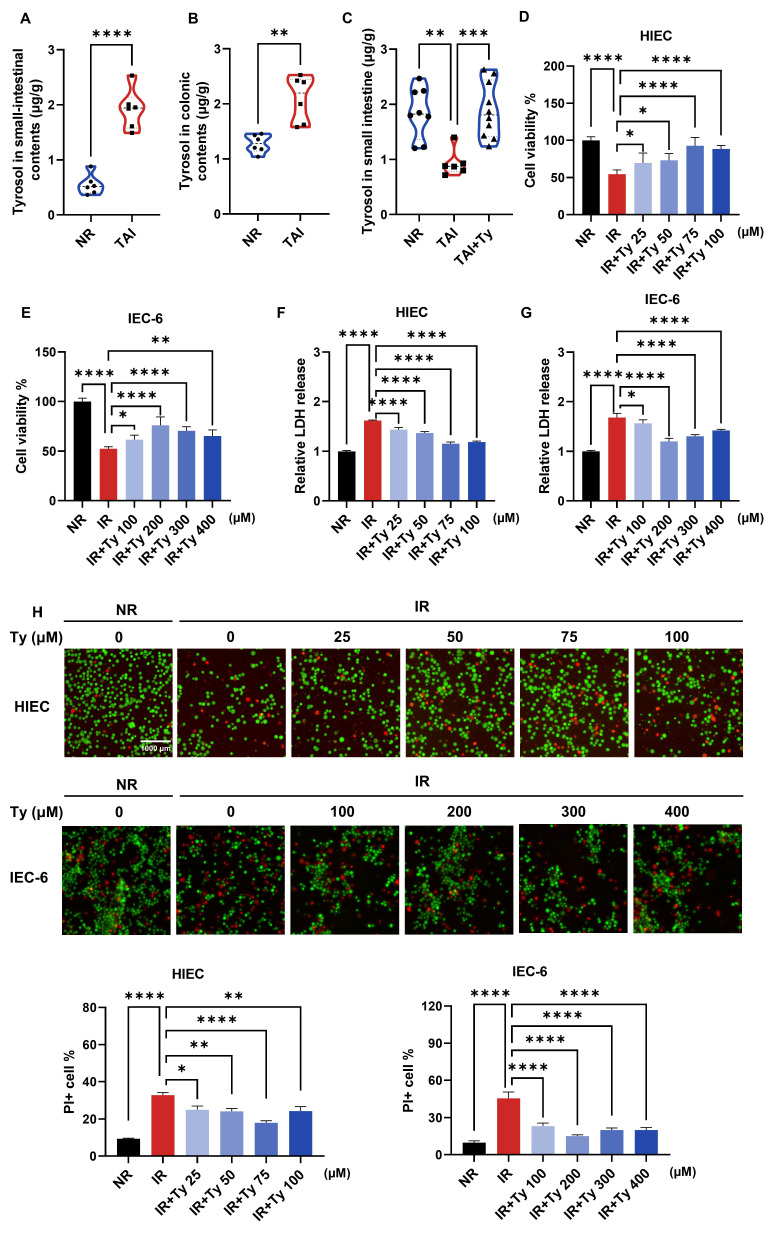
** Tyrosol supplementation mitigates RIII *in vitro*.** (A-C) Absolute quantification of tyrosol in mouse small-intestinal contents (A), colonic contents (B) and small-intestine (C), with each point representing an individual mouse. (D-E) CCK-8 assay of concentration gradient tyrosol treatment on irradiated HIECs (D) and IEC-6 cells (E). (F-G) LDH release assay of concentration gradient tyrosol treatment on irradiated HIECs (F) and IEC-6 cells (G). (H) Calcein-AM/PI staining of concentration gradient tyrosol treatment on irradiated HIECs and IEC-6 cells: Top, representative double-staining images; Bottom, quantification of PI-positive cells. All cells were pre-treated with tyrosol for 24 h prior to 8 Gy irradiation, and all indicators were detected at 72 h post-irradiation. Bars represent mean ± SD. Ty, tyrosol. *p < 0.05, **p < 0.01, ***p < 0.001, ****p < 0.0001.

To determine whether metabolic changes were consistent across irradiation models, we first quantified tyrosol in intestinal contents and tissues. Consistently, following TAI in mice, tyrosol levels increased in the luminal contents of both the small and large intestines, whereas tyrosol within small intestinal tissue decreased; exogenous supplementation restored tissue levels (Fig. 3A-C). We next investigated whether tyrosol protects against radiation-induced intestinal injury (RIII) using complementary *in vitro* and *in vivo* approaches. *In vitro* assessments demonstrated that tyrosol was non-cytotoxic up to concentrations of 100 μM in human intestinal epithelial cells (HIECs) and 400 μM in rat IEC-6 cells (Fig. S1C, D). Following irradiation, tyrosol significantly improved cell viability in a dose-dependent manner, as measured by Cell Counting Kit-8 (CCK8) assay (Fig. 3D, E), lactate dehydrogenase (LDH) release assay (Fig. 3F, G), and live/dead cell staining with Calcein-AM/PI (Fig. 3H). Optimal radioprotective doses of tyrosol at 75 μM for HIECs and 200 μM for IEC-6 cells, were applied for subsequent experiments.

**Figure 4:**
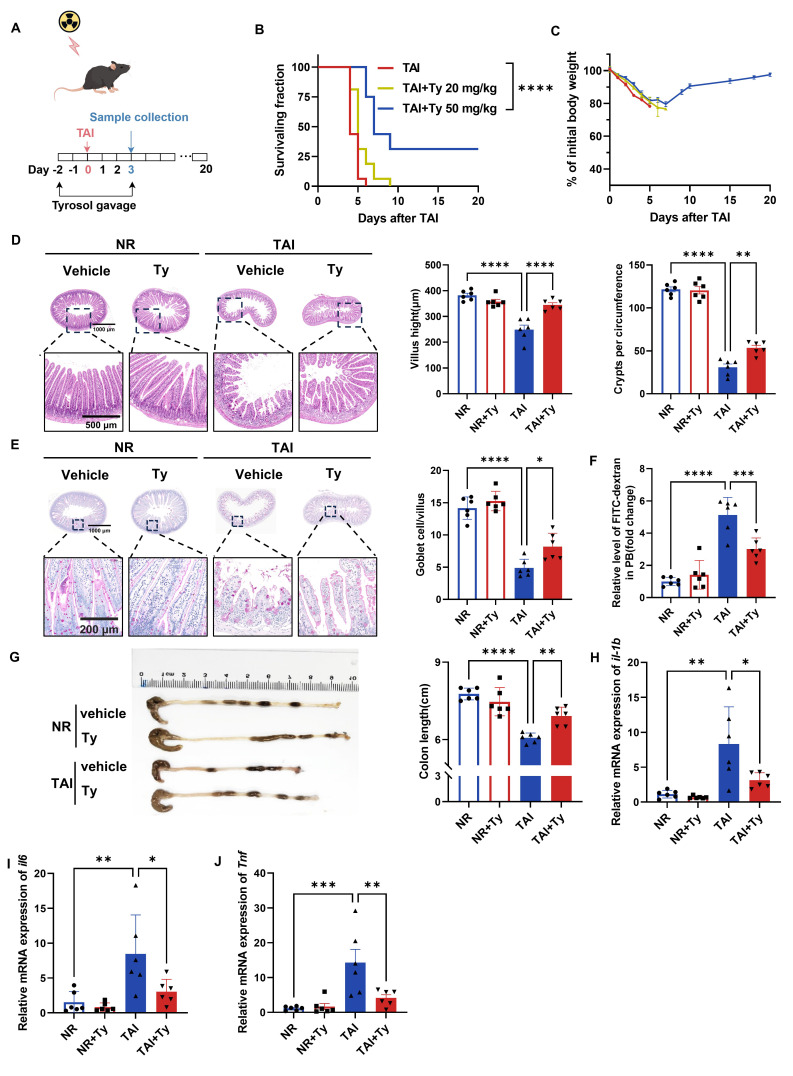
** Tyrosol supplementation mitigates RIII *in vivo*.** (A) Schematic of tyrosol administration (20 or 50 mg/kg) via gavage to ameliorate radiation-induced intestinal injury. Mice were monitored over 20 days or euthanized at day 3 post-12 Gy TAI. (B-C) Survival curves (B) and body weight changes (C) of irradiated mice. (n = 16 per group). (D) Representative H&E-stained intestinal sections at day 0 and 3 post-12 Gy TAI; right, quantification of villus height and crypt numbers per circumference. (E) Representative PAS-stained small intestine sections from vehicle- and tyrosol-treated mice at day 0 and 3 post-12 Gy TAI (scale bar = 200 μm); right, quantification of globet cells per villus. (F) Gut permeability assessed by serum fluorescence intensity at day 3, normalized to non-irradiated controls. (G) Colon lengths measured at day 3 post-TAI; left: representative images of colons. (H-J) qRT-PCR analysis of *il1b*(H),* il6*(I), and *Tnf* (J) expression in small intestinal tissues at day 3 post-TAI. Each symbol represents one mouse. Bars represent mean ± SD. Ty, tyrosol; TAI, total abdominal irradiation. *p < 0.05, **p < 0.01, ***p < 0.001, ****p < 0.0001.

*In vivo*, mice received daily oral tyrosol at 20 or 50 mg/kg for 2 days prior to 12 Gy TAI, continuing for 3 days post-irradiation (Fig. 4A). Although intervention with low-dose tyrosol (20 mg/kg) had no significant effects on mouse body weight and survival rate, high-dose tyrosol (50 mg/kg) significantly increased the survival rate of irradiated mice by 31.25% in comparison with no drug treatment control (Fig. 4B). Furthermore, by day 10 post-irradiation, the body weight of mice in the high-dose group had stabilized and exhibited with a gradual recovery trend (Fig. 4C). Histological analysis performed on day 3 post-TAI revealed that, in comparison with control, tyrosol at 50 mg/kg significantly preserved intestinal villus length and crypt density (Fig. 4D) and increased the number of goblet cells in the irradiated mice (Fig. 4E). FITC-dextran permeability assay confirmed a reduction in intestinal permeability in the tyrosol-treated mice (Fig. 4F). Tyrosol also attenuated colonic shortening after TAI (Fig. 4G). Meanwhile, qRT-PCR analysis demonstrated significant downregulation of proinflammatory cytokine genes (*il1b*,* il6*, *Tnf*) in the small intestine of irradiated mice (Fig. 4H-J). Collectively, these findings strongly support the potential of tyrosol as a safe and effective radioprotective agent.

Considering tyrosol derives from tyrosine metabolism—a component of amino acids with favorable safety profiles—we investigated whether exogenous tyrosine confers similar protection. Mice received 100 or 500 mg/kg L-tyrosine via drinking water for ten days prior to 12 Gy TAI, continuing until day four post-irradiation. Neither dose improved survival nor prevented weight loss (Fig. S1E, F). Furthermore, analysis of fecal and intestinal samples revealed that exogenous L-tyrosine did not significantly increase endogenous tyrosol levels (Fig. S1G, H), suggesting limited conversion or bioavailability.

### Tyrosol mitigates radiation-induced ER stress via SCD1

**Figure 5:**
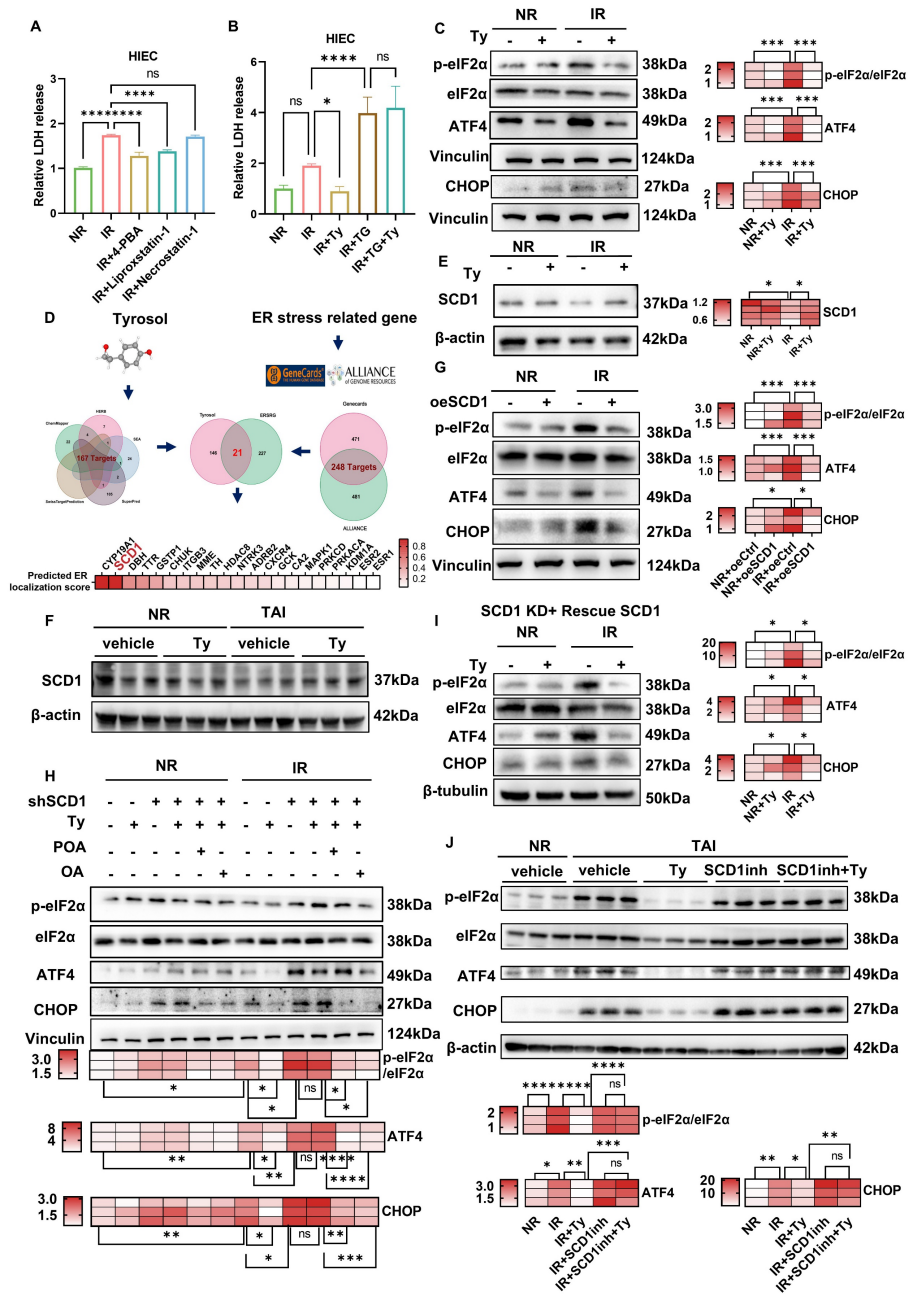
** Tyrosol mitigates radiation-induced ER stress in a SCD1-dependent manner.** (A) LDH release assay of HIECs pretreated with 4-PBA (ER stress inhibitor), Liproxstatin-1 (ferroptosis inhibitor), or Necrostatin-1 (necroptosis inhibitor), detected at 72 h post-8 Gy IR. (B) LDH release assays of HIECs pretreated with tyrosol combined with TG, assessed at 72 h post-8 Gy IR. (C) Western blot analysis of p-eIF2α, eIF2α, ATF4, and CHOP at 12 h post-IR in tyrosol-pretreated HIECs; right, quantification normalized to vinculin (relative to NR). (D) Network pharmacology analysis of tyrosol's potential targets involved in ER stress alleviation. (E) Western blot analysis of SCD1 in tyrosol-pretreated HIECs at 12 h post-IR; right: quantification of SCD1 normalized to β-actin relative to non-irradiated controls. (F) Western blot analysis of SCD1 in mouse small intestines at 12 h after 12 Gy TAI. (G) Western blot analysis of p-eIF2α, eIF2α, ATF4, and CHOP at 12 h post-IR in HIECs overexpressing SCD1 or vector (oeCtrl); right, quantification normalized to vinculin (relative to NR). (H) Western blot analysis of p-eIF2α, eIF2α, ATF4, and CHOP in HIECs treated with tyrosol, OA, or POA, following transfection with either shSCD1 or non-targeting shRNA (shNC); bottom, quantification normalized to vinculin (relative to NR). (I) Western blot analysis of p-eIF2α, eIF2α, ATF4, and CHOP in HIECs transfected with wild-type SCD1 and treated with tyrosol prior to IR; right, quantification normalized to β-tubulin (relative to NR). (J) Western blot analysis of p-eIF2α, eIF2α, ATF4, and CHOP in mouse small intestines (five groups, 12 h post-12 Gy TAI); bottom, quantification normalized to β-actin (relative to NR). Bars represent mean ± SD. IR, irradiation; Ty, tyrosol; 4-PBA, 4-phenylbutyric acid; OA, oleic acid; POA, palmitoleic acid; SCD1inh, SCD1 inhibitor (A939572). *p < 0.05, **p < 0.01, ***p < 0.001, ****p < 0.0001, ns, no significant difference.

To elucidate the mechanism by which tyrosol mitigates RIII, we evaluated the roles of ER stress, ferroptosis, and necroptosis in irradiated HIECs. Pharmacological inhibition of ER stress by 4-PBA and inhibition of ferroptosis by Liproxstatin-1, but not necroptosis, significantly attenuated radiation-induced cytotoxicity (Fig. 5A). Given that Liproxstatin-1 exerts its effects mainly by restoring intracellular glutathione (GSH) levels, we investigated the impact of tyrosol on GSH/GSSG ratios following irradiation. The results demonstrated a significant decrease in GSH/GSSG ratio post-irradiation, however, tyrosol treatment did not reverse this alteration (Fig. S2A). We therefore focused subsequent mechanistic studies on ER stress.

To define the role of ER stress in radiation injury and determine whether its modulation mediates tyrosol's radioprotection, we combined ER stress modulators with an ER stress reporter in irradiated intestinal epithelial cells. ER stress inhibitors 4-PBA and TUDCA dose-dependently alleviated radiation-induced cellular damage (Fig. S2B-E), indicating that ER stress contributes to radiation injury and that its inhibition protects intestinal epithelial cells. Consistently, tyrosol markedly reduced radiation-induced damage in both cell types, but co-treatment with 4-PBA or TUDCA provided no additional benefit (Fig. S2F-I). Tyrosol alone also significantly suppressed ER stress reporter activity (Fig. S2K). Importantly, thapsigargin (TG), an ER stress inducer, abrogated tyrosol's protective effect (Fig. 5B; Fig. S2J). Collectively, these data support ER stress suppression as a key mechanistic basis for tyrosol-mediated radioprotection.

To gain deeper insights into how tyrosol regulates ER stress, we analyzed the expression levels of key proteins involved in major ER stress signaling pathways. As shown in Supplementary Fig. S2L, exposure of HIECs to 8 Gy irradiation led to a significant increase in the levels of ER stress markers at 12 h post-irradiation, including the phosphorylated eukaryotic initiation factor 2α (p-eIF2α)/total eIF2α ratio, activating transcription factor 4 (ATF4), and C/EBP homologous protein (CHOP). Notably, tyrosol treatment markedly suppressed these elevations (Fig. 5C), suggesting the role of tyrosol in mitigating radiation-triggered ER stress. Network pharmacology analysis identified 21 shared targets between tyrosol targets (167 candidates) and ER stress-related genes (248 candidates) (Fig. 5D). Among these, SCD1, an ER-resident enzyme critical for catalyzing Δ9-desaturation of saturated fatty acids (SAFs, e.g., stearic acid) to MUFAs (e.g., oleic acid (OA) and palmitoleic acid (POA)) 30, was identified as a key mediator of tyrosol's effects. Western blot analysis revealed that SCD1 expression was downregulated post-irradiation, and this decrease was reversed by tyrosol treatment in both irradiated HIECs and mouse intestines (Fig. 5E, F). To elucidate the involvement of SCD1, we modulated its expression in HIECs before irradiation (Supplementary Fig. S3A-C). SCD1 overexpression sharply blunted the radiation-induced surge in p-eIF2α/eIF2α, ATF4, and CHOP, whereas SCD1 downregulation intensified these ER-stress hallmarks and abrogated tyrosol's radioprotective effect (Fig. 5G, H). Moreover, restoration of the lost enzymatic activity, either by supplementing with SCD1's catalytic substrates of oleic acid (OA) and palmitoleic acid (POA), or reintroducing wild-type SCD1 into cells, fully reinstated tyrosol's capacity to suppress ER stress (Fig. 5H, I). Thus, convergent lines of gain- and loss-of-function data established SCD1 as the unique, indispensable mediator through which tyrosol preserved ER homeostasis. Critically, in the irradiated mice, tyrosol potently suppressed radiation-induced upregulation of ER-stress markers, and this radioprotection was entirely reversed by co-administration of a selective SCD1 inhibitor A939572 (Fig. 5J). Taken together, these findings demonstrated that tyrosol attenuated radiation-induced ER stress in an SCD1-dependent manner.

### Tyrosol alleviates RIII in a SCD1-dependent manner

**Figure 6:**
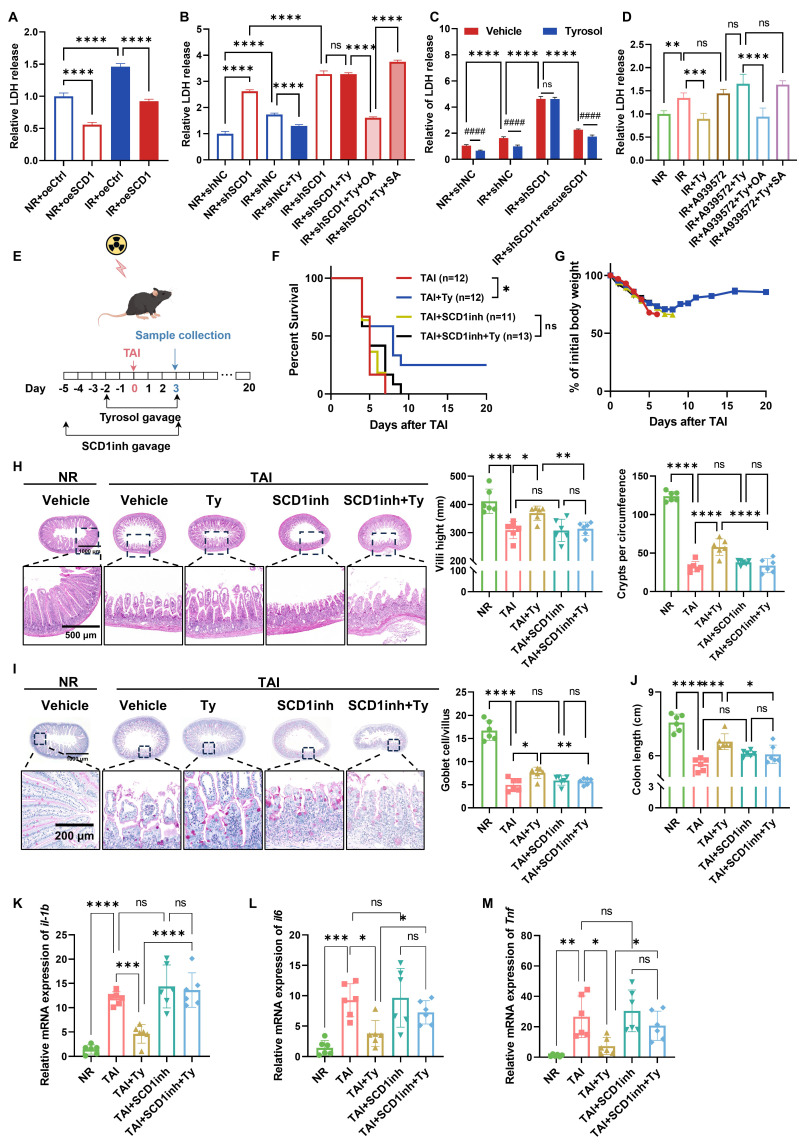
**Tyrosol mitigates RIII in a SCD1-dependent manner.** (A) LDH release assay of SCD1-overexpressing HIECs at 72 h after 8 Gy IR. (B) LDH release assay of SCD1-depleted HIECs pretreated with tyrosol combined with OA or SA, assessed at 72 h post-IR. (C) LDH release assay of SCD1-depleted HIECs pretreated with tyrosol alongside SCD1 rescue or without, evaluated at 72 h after 8 Gy IR. (D) LDH release assays of HIECs pretreated with tyrosol combined with A939572, OA, or SA, assessed at 72 h post-8 Gy IR. (E) Schematic of *in vivo* treatment: tyrosol (50 mg/kg) gavage combined with SCD1 inhibitor (A939572, 30 mg/kg) intraperitoneal injection, with irradiated mice monitored over 20 days or euthanized on day 3 post-TAI. (F-G) Survival curves (F) and body weight changes (G) of irradiated mice over the observation period. (H-I) Representative H&E (H) and PAS (I) stained intestinal sections at day 0 and 3 post-12 Gy TAI; right, quantification of villus height, crypt numbers (H) and goblet cell count per villus (I). (J) Colon length of mice sacrificed on day 3 after 12 Gy TA. (K-M) qRT-PCR analysis of *il1b* (K), *il6* (L), and *Tnf* (M) expression in small intestine tissues at day 3 post-12 Gy TAI. Each symbol represents one mouse. Bars represent mean ± SD. IR, irradiation; Ty, tyrosol; SCD1inh, SCD1 inhibitor (A939572); OA, oleic acid; POA, palmitoleic acid; SA, stearic acid; PA, palmitic acid. *p < 0.05, ***p < 0.001, ****p < 0.0001, ####*p* < 0.0001, ns, no significant difference.

To further validate the role of SCD1 in RIII and confirm its function as a critical target for tyrosol-mediated radioprotection, we further performed complementary *in vitro* and *in vivo* experiments. Genetic manipulation of SCD1 verified its critical involvement: LDH release assays indicated that SCD1 overexpression mitigated radiation-induced cytotoxicity in HIECs (Fig. 6A), whereas SCD1 knockdown exacerbated radiation injury and abolished the radioprotective benefits of tyrosol (Fig. 6B). Supplementation with exogenous OA, the enzymatic product of SCD1, restored tyrosol's radioprotection in SCD1-deficient cells. In contrast, stearic acid (SA), the substrate of SCD1, had no such radioprotective effect (Fig. 6B). Reintroduction of wild-type SCD1 into SCD1-knockdown cells fully rescued the efficacy of tyrosol (Fig. 6C). To further validate SCD1 as a functional target, we combined SCD1 inhibitors (A939572 or PluriSln #1) with tyrosol in HIEC and IEC-6 cells. Dose-finding studies with LDH assays identified the maximal inhibitor concentrations that did not induce additional radiation damage (Fig. S3D-G). Co-treatment with tyrosol and SCD1 inhibitors abolished tyrosol's radioprotective effect; however, exogenous OA supplementation rescued this protection during SCD1 inhibition, whereas SA remained ineffective (Fig. 6D; Fig. S3H-J).

Consistently, we also demonstrated that the radioprotective effect of tyrosol against RIII in animal models was SCD1-dependent. Administration of SCD1 inhibitor A939572 reversed the survival benefits and body weight preservation conferred by tyrosol in the irradiated mice (Fig. 6E-G). Histological analyses further showed that the SCD1 inhibitor fully abrogated tyrosol's protective actions, such as maintenance of intestinal villus length, crypt count, and goblet cell count (Fig. 6H, I), and attenuation of colonic shortening (Fig. 6J). Additionally, SCD1 inhibition abrogated tyrosol's ability to suppress radiation-induced upregulation of proinflammatory cytokine genes of *Il1b*, *Il6*, and *Tnf* (Fig. 6K-M).

These findings establish SCD1 as the central mediator of tyrosol's radioprotection, with its enzymatic activity supporting tyrosol's effects on mitigating radiation-induced intestinal epithelial cell cytotoxicity and suppressing proinflammatory cytokine upregulation.

### Tyrosol inhibits VCP-mediated ubiquitin-dependent degradation of SCD1

**Figure 7:**
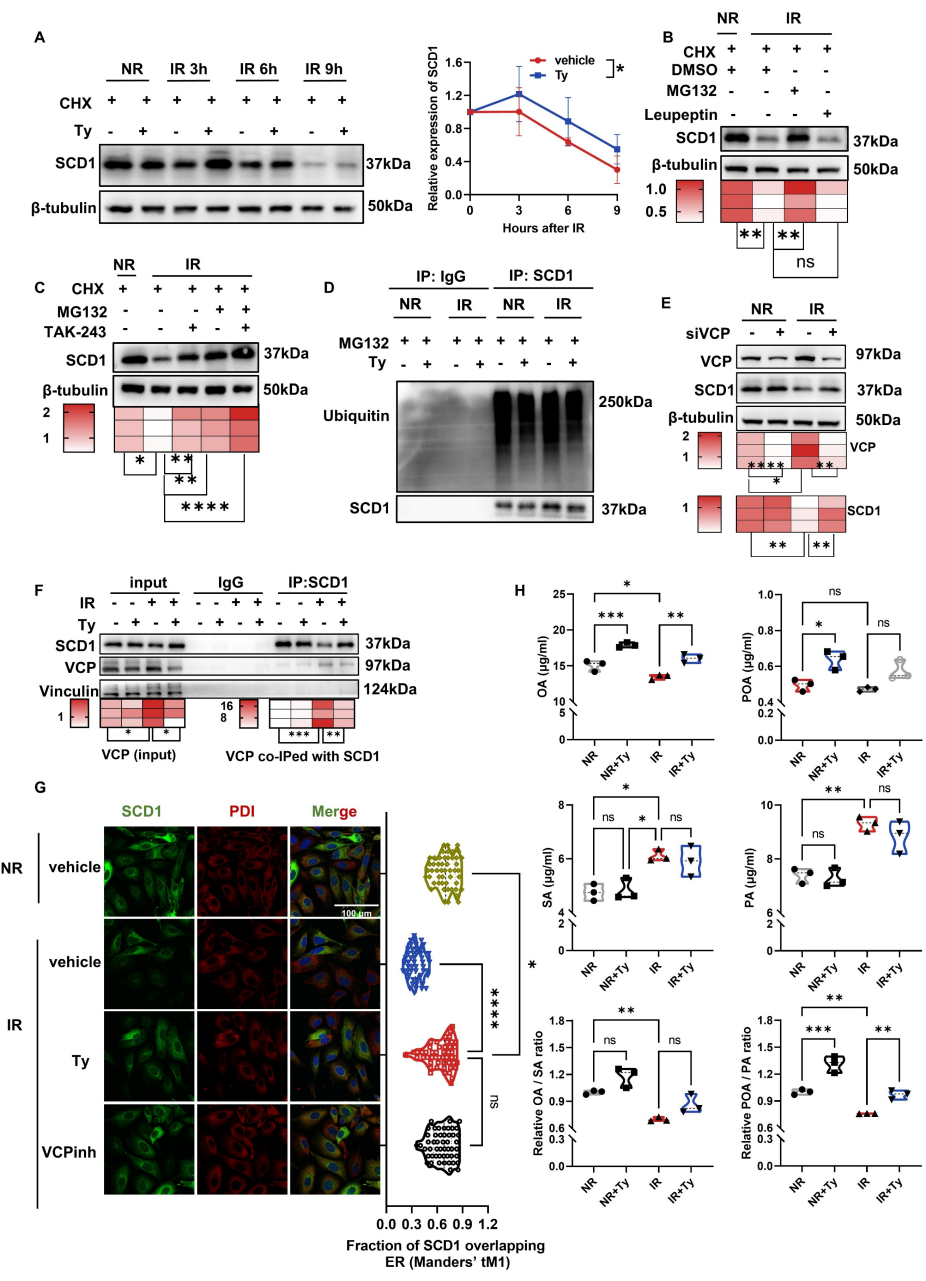
** Tyrosol inhibits VCP-mediated degradation of SCD1 in HIECs.** (A) Western blot analysis of SCD1 levels in the irradiated HIECs treated with tyrosol at indicating time points after IR; cells pretreated with CHX for 2 h prior to IR; right: quantification of SCD1 relative to β-tubulin, normalized to 0 h. (B) Western blot analysis of SCD1 levels at 12 h post-IR following treatment with CHX plus MG132 or Leupeptin in HIECs; bottom, quantification normalized to β-tubulin (relative to NR). (C) Western blot analysis of SCD1 at 12 h post-IR after CHX with MG132 or TAK-243; bottom, quantification normalized to β-tubulin (relative to NR). (D) Co-IP showing ubiquitination of SCD1 at 12 h post-IR, with MG132 and tyrosol pretreatment. (E) Western blot analysis of VCP and SCD1 levels at 12 h post-IR in HIECs transfected with VCP siRNA or control siRNA; bottom, quantification normalized to β-tubulin (relative to NR). (F) Co-IP demonstrating interaction between SCD1 and VCP with or without 24 h tyrosol treatment prior to IR; bottom, quantification normalized to vinculin (relative to NR). (G) Immunofluorescence staining of SCD1 and PDI, following 24 h tyrosol or 2 h VCP inhibitor (NMS-873) pretreatment before IR (8 Gy). Scale bar = 20 μm. Right: Manders' coefficient-based quantification of SCD1-ER colocalization in HIECs, 50 cells per group. (H) Violin plots presenting SCD1 metabolic products (OA, POA) and substrates (SA, PA) measured by LC-MS in HIECs pretreated with tyrosol prior to IR (n=3 per group). Bars represent mean ± SD. IR, irradiation; Ty, tyrosol; CHX, cycloheximide; SA, stearic acid; OA, oleic acid; PA, palmitic acid; POA, palmitoleic acid; VCPinh, VCP inhibitor. *p < 0.05, **p < 0.01, ***p < 0.001, ****p < 0.0001, ns, no significant difference.

With SCD1 validated as tyrosol's core radioprotective mediator, we further explored how tyrosol maintains SCD1 protein levels post-irradiation. Analysis of qRT-PCR revealed no significant effect of tyrosol on SCD1 mRNA expression under either irradiated or non-irradiated conditions (Fig. S4A, B), indicating that tyrosol regulates SCD1 stability at the post-transcriptional level. Given that radiation-induced ER stress can accelerate protein degradation via the ubiquitin-proteasome system (UPS) or autophagy-lysosome pathway, we hypothesized that tyrosol stabilizes SCD1 protein by inhibiting its degradation. Cycloheximide chase assay confirmed that tyrosol delayed the degradation of SCD1 (Fig. 7A), supporting the notion that tyrosol maintained SCD1 protein level by inhibiting its degradation.

Further experiments demonstrated that pharmacological inhibition of proteasome with MG132 rescued radiation-induced SCD1 protein loss, whereas inhibition of lysosomes with Leupeptin had no such effect (Fig. 7B), implicating that the proteasomal degradation pathway mediated SCD1 reduction after radiation. Consistent with this, treatment of cells with TAK-243, a ubiquitin kinase inhibitor, also preserved SCD1 protein level (Fig. 7C), suggesting the involvement of a ubiquitin-dependent mechanism. Unexpectedly, co-immunoprecipitation (co-IP) assay revealed that there was no significant change in SCD1 ubiquitination level following irradiation (Fig. 7D), indicating that SCD1's degradation post-irradiation was not due to the increased SCD1 ubiquitination.

Given that valosin-containing protein (VCP) functions in shuttling ubiquitinated proteins to the proteasome and interacts with SCD1 51, 52, we investigated whether VCP is involved in radiation-induced SCD1 degradation in HIECs. Using VCP-specific inhibitor or siRNA, we found that VCP inhibitor suppressed radiation-induced SCD1 degradation in a dose-dependent manner (Fig. S4C). Similarly, VCP siRNA prevented post-irradiation downregulation of SCD1 protein without promoting SCD1 transcription (Fig. 7E; Fig. S4D). Co-IP assay revealed that the interaction between VCP and SCD1 and the total VCP protein levels were increased following irradiation, which were significantly attenuated by tyrosol (Fig. 7F). Consistently, irradiation-induced upregulation of VCP protein in the small intestine was reversed by tyrosol treatment (Fig. S4E). Moreover, immunofluorescence staining showed that both VCP inhibition and tyrosol treatment maintained SCD1 levels and enhanced its localization to ER (Fig. 7G; Fig. S4F). These findings demonstrated that VCP mediated the irradiation-enhanced SCD1 proteasomal degradation, and tyrosol counteracted this process to preserve SCD1 stability.

As a key enzyme regulating lipid homeostasis, SCD1 converts the saturated fatty acids (SFAs) (e.g. SA and palmitic acid (PA)) to MUFAs (e.g. OA and POA). Since enzyme abundance directly modulates its activity 53, we subsequently focused on the levels of these SCD1-related fatty acids. Consistent with the earlier observed changes in SCD1 protein levels, lipid metabolism assay revealed that radiation decreased SCD1-derived MUFAs (OA and POA) while increased the levels of its SFAs substrates (SA and PA). In contrast, tyrosol treatment restored the ratios of OA/SA, particularly POA/PA (Fig. 7H). Collectively, tyrosol stabilized SCD1 protein by disrupting VCP-mediated proteasomal trafficking of SCD1, thereby preserving both SCD1 protein abundance and its enzymatic activity.

### Tyrosol binds and activates SCD1 to facilitates MUFA synthesis

**Figure 8:**
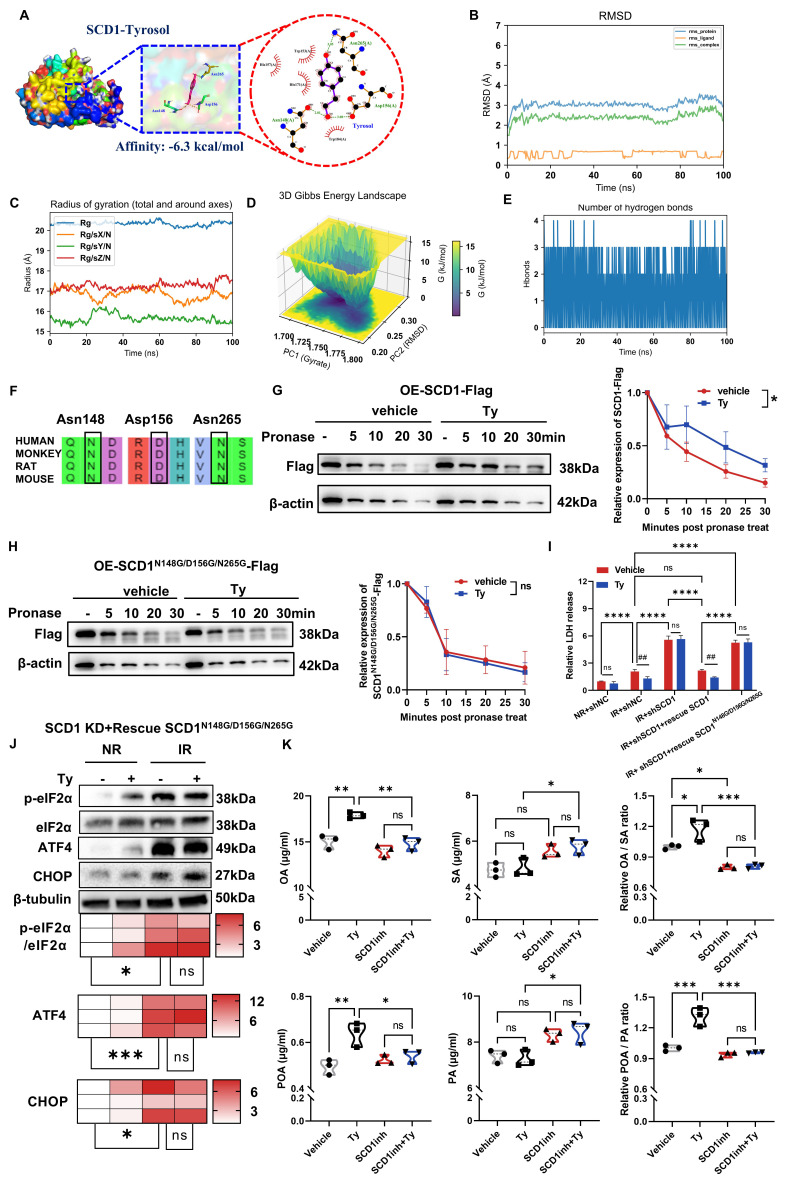
** Tyrosol binds to SCD1 and enhances its enzymatic activity.** (A) Surface representation of the docking model showing the interface residues between SCD1 and tyrosol; tyrosol in purple, amino acid residues in yellow, hydrogen bonds indicated by green dotted lines. The binding energy is -6.3 kcal/mol. (B) The RMSD values for SCD1-tyrosol complex. (C) The Radius of gyration for SCD1-tyrosol complex. (D) Three-dimensional mappings of the free energy landscape. (E) Dynamics of hydrogen bonding observed in the molecular dynamic simulations. (F) Phylogenetic conservation of SCD1 amino acid sequences across humans, monkeys, rats, and mice. (G-H) DARTS assay immunoblots of HIECs transfected with wild-type (G) or N148G/D156G/N265G mutant (H) SCD1-Flag constructs; right, quantification normalized to β-actin (relative to non-pronase treatment). (I) LDH release assay of HIECs pretreated with tyrosol, evaluated at 72 h post IR; includes cells with SCD1 depletion transfected with either wild-type or mutant SCD1 constructs. (J) Western blot analysis of p-eIF2α, eIF2α, ATF4, and CHOP in HIECs transfected with SCD1 mutant constructs (N148G, D156G, N265G) and treated with tyrosol prior to IR; bottom, quantification normalized to β-tubulin (relative to NR). (K) Violin plots of SCD1 catalytic products (OA, POA) and substrates (SA, PA) measured by LC-MS following treatment with tyrosol, SCD1 inhibitor (SCD1inh), or combination (n=3 per group). Bars represent mean ± SD. IR, irradiation; RMSD: root-mean-square deviation; Ty, tyrosol; SA, stearic acid; OA, oleic acid; PA, palmitic acid; POA, palmitoleic acid. *p < 0.05, **p < 0.01, ***p < 0.001, ****p < 0.0001, ##*p* < 0.01, ns, no significant difference.

Since the network pharmacology analysis has identified SCD1 as a target of tyrosol (Fig. 5D), we wonder to know how they interact each other. Molecular docking analysis demonstrated that tyrosol was capable of binding to SCD1 with a binding affinity of -6.3 kcal/mol, a value that indicated a strong intermolecular interaction between these two molecules (Fig. 8A). Further validation via molecular dynamics (MD) simulations showed that the root-mean-square deviation (RMSD) of the SCD1-tyrosol system quickly plateaued within 10 ns remained stable for the entire duration of the simulation. This observation confirmed the formation of a relatively stable SCD1-tyrosol complex (Fig. 8B). Analysis of the radius of gyration (Rg) revealed that the system maintained a compact, isotropic, and stable conformation throughout the 100 ns trajectory, with no significant structural perturbations, verifying the full convergence of the MD simulation (Fig. 8C). Additionally, Gibbs free energy landscape analysis identified a distinct low-energy region for the SCD1-tyrosol complex (Fig. 8D), which provides further evidence to validate the stable binding between SCD1 and tyrosol.

Hydrogen bonds are the primary chemical interactions mediating drug molecule-protein binding. MD simulation results showed that the number of hydrogen bonds between tyrosol and SCD1 mainly fluctuated between 2 and 3, with a high occupancy rate throughout the simulation (Fig. 8E), indicating that hydrogen bonds are the dominant force stabilizing their binding. Analysis of the optimal binding mode from molecular docking demonstrated that tyrosol could form hydrogen bonds with the Asn148, Asp156, and Asn265 residues of SCD1 (Fig. 8A). Notably, these amino acid sites are highly conserved across human, cynomolgus monkey, rat, and mouse (Fig. 8F), supporting their role as key tyrosol-binding sites on SCD1. This was further validated by DARTS experiments, which showed that tyrosol increased the proteolytic resistance of wild-type SCD1 but not of SCD1 with mutations at these three sites (Fig. 8G, H), confirming these residues as critical for tyrosol binding.

Further functional assays demonstrated that reintroduction the SCD1 mutant (N148G/D156G/N265G) into HIECs neither attenuated radiation-induced cytotoxicity nor enhanced the radioprotective effect of tyrosol (Fig. 8I; Fig. S3C). Consistently, SCD1^N148G/D156G/N265G^ failed to restore tyrosol's ability to suppress ER stress-related proteins, underscoring the critical role of tyrosol-SCD1 binding in mediating tyrosol's radioprotective effects (Fig. 8J).

To determine whether the SCD1-tyrosol interaction modulates SCD1's enzymatic activity, we performed targeted metabolomics analysis. Results showed that tyrosol significantly increased the levels of OA and POA that serve as SCD1's catalytic substrates. While it caused minimal alterations to the level of its substrate (SA and PA), thereby elevating the ratios of OA/SA and POA/PA by 1.2- and 1.3-fold, respectively (Fig. 8K). Critically, pretreatment of cells with A939572, a selective SCD1 inhibitor, completely abrogated these metabolic effects of tyrosol. This finding further confirmed that tyrosol regulated cellular lipid metabolism through SCD1, underscoring that SCD1 is a direct functional target of tyrosol. Taken together, these findings demonstrated that tyrosol exerted radioprotective effects on intestinal epithelial cells by directly binding to and activating SCD1, thereby promoting the biosynthesis of MUFAs.

## Discussion

Current research highlights gut-derived metabolites as promising therapeutic targets for various diseases, especially gastrointestinal (GI) disorders, and their potential relevance to radiation-induced intestinal injury (RIII), a major complication of radiotherapy. In parallel, interest in phenolic compounds generated through gut microbiota metabolism has expanded rapidly 11, 54, advancing our understanding of intestinal metabolites and solidifying this compound class as a focal point in microbiome research. Our untargeted metabolomic analysis of irradiated samples identified two key observations: significant enrichment of the fecal tyrosine metabolic pathway and elevated levels of the phenolic compound tyrosol. When combined with subsequent functional experimental findings, these results suggest an adaptive response in irradiated organisms, wherein elevated tyrosol levels mediate endogenous intestinal protection. Although small intestinal epithelial cells exhibit relatively robust renewal capacity, radiation-induced disruption of the intestinal mucosal barrier in the early post-irradiation days remains a primary driver of fatal complications 55. In this study, we demonstrated that tyrosol directly mitigated radiation-induced epithelial injury, enhanced intestinal barrier integrity, and conferred immediate protection against such damage. Furthermore, converging *in vitro* and *in vivo* evidence establishes tyrosol's dual functional properties: it preserves intestinal epithelial architecture while suppressing the production of proinflammatory cytokines. Mechanistically, this dual action parallels that of well-characterized phenolic compounds such as hydroxytyrosol and resveratrol 56, 57.

The pathogenesis of RIII involves multiple interconnected mechanisms, with endoplasmic reticulum (ER) stress serving as a central mediator 58. The ER is vital for protein folding, lipid biosynthesis, and calcium homeostasis 59. Upon irradiation, oxidative stress, protein misfolding, and disturbed lipid metabolism converge to activate ER stress and the unfolded protein response (UPR) 60-62. Although the UPR is initially adaptive, the prolonged or excessive ER stress favors apoptotic signaling 63, 64, thereby promoting epithelial cell loss and compromising the mucosal barrier. Accordingly, ER stress has become an attractive therapeutic target; modulating regulators such as β-arrestin1 or PERK can dampen ER stress and facilitate intestinal stem cell proliferation and tissue repair after irradiation 58. In line with this framework, our data indicate that tyrosol interrupts this pathological cascade by directly relieving ER stress downstream of the upstream insults.

High-dose radiation impairs gut epithelial barrier integrity and triggers hyperactive immune responses, perpetuating a self-amplifying cycle of progressive tissue damage 65. In contrast to SCFAs, which mainly exert protective effect by enhancing epithelial renewal 66 and modulating immune responses 67, tyrosol operates via a distinct mechanism: it stabilizes SCD1 protein and enhances its enzymatic activity. This dual action restores lipid homeostasis within intestinal epithelial cells, alleviates ER stress, and preserves cellular proteostasis, thereby maintaining epithelial integrity and suppressing inflammatory cascades. Mechanistically, tyrosol enhances radioprotective effect via dual regulation of SCD1: it suppresses VCP-mediated SCD1 protein degradation and enhances SCD1's catalytic activity through direct binding to three conserved amino acid residues (Asn148, Asp156, Asn265). This coordinated dual action promotes the biosynthesis of MUFAs, a process critical for maintaining membrane fluidity and sustaining ER function, ultimately mitigating ER stress 33, 68. Collectively, this dual regulatory mechanism (enzyme activation + proteostatic stabilization) provides a novel mechanistic insight into how gut-derived metabolites modulate proteostasis pathways, distinct from conventional approaches that focus solely on regulating protein expression.

Current clinically used conventional radioprotectors not only exhibit poor patient tolerance but also, critically, lack tumor selectivity. This limitation may compromise radiotherapy efficacy and even promote tumor progression 69. Notably, recent studies have confirmed that tyrosol exhibits selective antitumor activity against colorectal cancer cells (e.g., MC38) in both *in vitro* and *in vivo* models, while sparing the viability of non-malignant epithelial cells 23. Based on our experimental results, tyrosol shows significant potential to overcome the fundamental limitation through its dual-selective properties. Furthermore, our data together with extensive preclinical evidence indicate that tyrosol has low toxicity and favorable bioavailability 70. Although further studies are required to optimize its dosage and explore its interactions with tumor microenvironment, these findings establish tyrosol as a unique gut-derived agent with dual-selective properties, which holds translational potential for the treatment of gastrointestinal malignancies.

Beyond tyrosol's dual selectivity, our findings demonstrated that stabilization and activation of the SCD1-MUFA pathway can effectively mitigate radiation-induced intestinal epithelial damage, highlighting the therapeutic potential of targeting SCD1 and enhancing MUFA biosynthesis, specifically oleic acid and palmitoleic acid, for radiation protection. Future work may leverage the structural basis of tyrosol's binding to SCD1 to design small-molecule SCD1 agonists, which could serve as novel therapeutics, particularly for patients suffering from gut dysbiosis. However, this approach still presents notable challenges. In many cancers, including colorectal and other gastrointestinal malignancies, SCD1 is frequently upregulated 71. This upregulation supports tumor cell proliferation and survival through MUFA production, raising concerns that systemic activation of SCD1 during radiotherapy might inadvertently promote residual tumor growth. Therefore, the safety of SCD1 agonists in the cancer setting must be carefully considered. It may be prudent to employ such agents during post-surgical radiotherapy phases, when the primary tumor burden has been removed, to prevent radiation-induced enteritis without risking tumor progression. In addition, developing MUFA-based radioprotective strategies, such as dietary supplementation with MUFA-rich foods (e.g., high-oleic acid diets), warrants exploration through further *in vivo* and clinical studies. These approaches could offer a practical and safe adjunct or alternative to pharmacological SCD1 activation.

Given the well-documented antioxidant effects of phenolic compounds, future studies should evaluate whether tyrosol exerts similar antioxidant effects in the context of radiation, including its potential impacts on DNA damage repair pathways. Such insights could further elucidate its multifaceted role in radioprotection and inform the development of integrated therapeutic strategies.

## Conclusions

This study was the first to identify gut microbiota-derived tyrosol as a potent radioprotective agent in RIII via the novel tyrosol/SCD1/MUFA/ER stress axis. Mechanistically, tyrosol interacts with SCD1 by binding its specific residues (Asn148, Asp156, Asn265) and inhibiting VCP-mediated proteasomal degradation of SCD1, thereby enhancing SCD1 stability and increasing its enzymatic activity. It seems that tyrosol is an active repair factor for intestinal epithelium, although how tyrosol production is regulated by gut microbiota needs further research. Exogenous application of tyrosol could relieve RIII by preserving mucosal structure and suppressing ER stress, while SCD1 ablation abolishes tyrosol's protective effects. These findings provide targets (tyrosol, SCD1) for new therapeutic and preventative strategies for radiation-induced intestinal syndrome.

## Supplementary Material

Supplementary figures and tables.

## Figures and Tables

**Figure 9:**
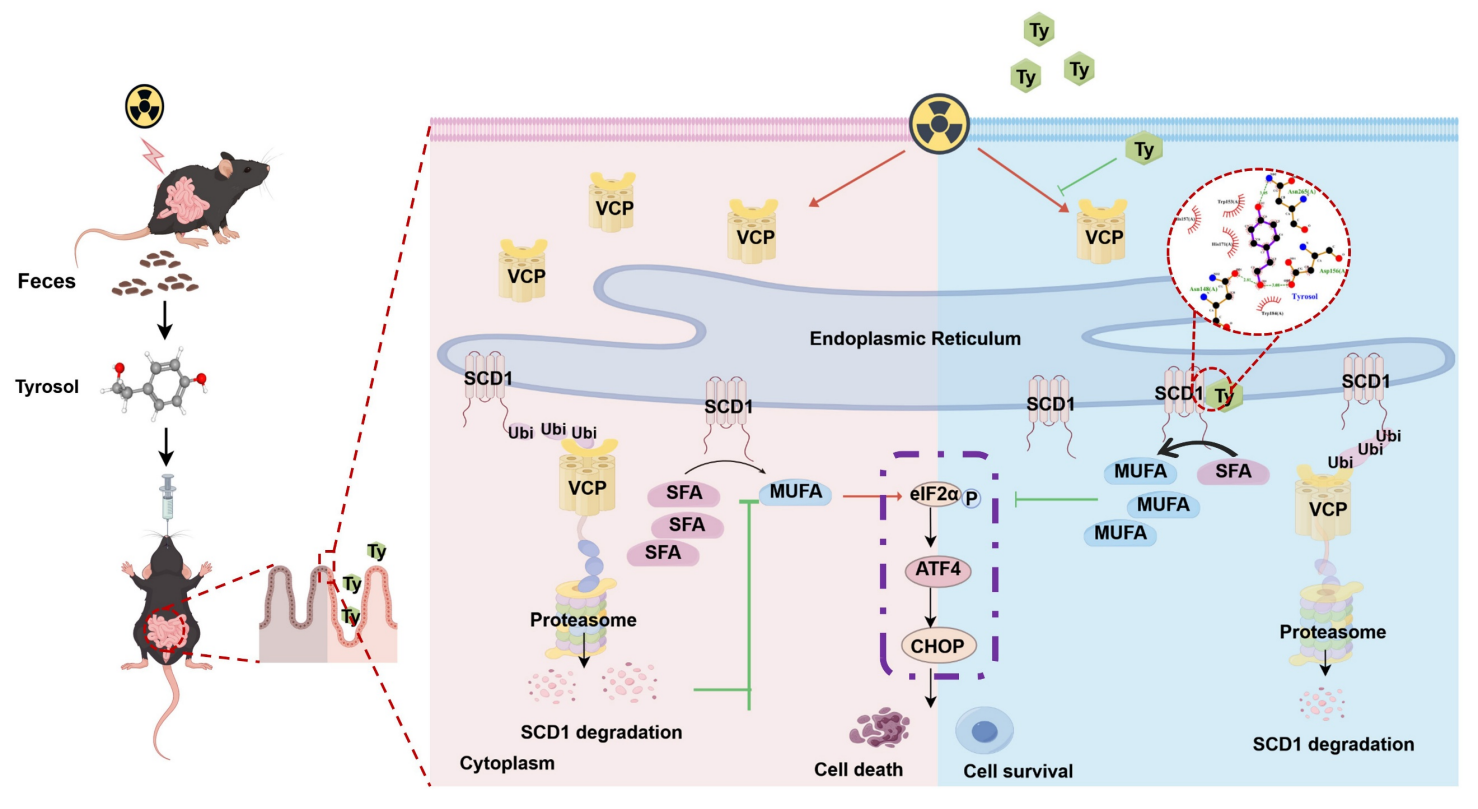
** Schematic model of tyrosol's protective effect against radiation-induced intestinal injury.** Fecal-derived tyrosol from mice directly binds to SCD1 in intestinal epithelial cells, enhancing its enzymatic activity and MUFAs (OA and POA) synthesis. This shift reduces endoplasmic reticulum stress induced by IR and alleviates intestinal injury. Radiation exposure increases VCP levels, facilitating the ubiquitination and proteasomal degradation of SCD1. Tyrosol maintains SCD1 stability by downregulating VCP post-IR, thereby mitigating intestinal damage. Abbreviations: IR, irradiation; Ty, tyrosol; MUFA, monounsaturated fatty acid; OA, oleic acid; POA, palmitoleic acid.

## Data Availability

The data from this study can be obtained by contacting the corresponding author with a reasonable request.

## Abbreviations

IR: Ionizing Radiation
RIII: Radiation-induced intestinal injury
Ty: Tyrosol
SCD1: Stearoyl-CoA desaturase 1
Asn: Asparagine
Asp: Aspartic acid
VCP: Valosin-containing protein
MUFA: Monounsaturated fatty acids
ER: Endoplasmic reticulum
p-eIF2α: Phosphorylated eukaryotic initiation factor 2α
ATF4: Activating transcription factor 4
CHOP: C/EBP homologous protein
SCFAs: Short-chain fatty acids
GI: Gastrointestinal
HIEC: Human intestinal epithelial cell
IEC-6: Intestinal epithelioid cell
HEK-293T: Human embryonic kidney 293T
CCK-8: Cell Counting Kit-8
LDH: Lactate dehydrogenase
PI: Propidium Iodide
H&E: Hematoxylin and eosin
PAS: Periodic acid-Schiff
ALT: Aminotransferase
AST: Aspartate aminotransferase
TBIL: Total bilirubin
BUN: Blood urea nitrogen
CREA: Creatinine
UA: Uric acid
NADH: Reduced Nicotinamide Adenine Dinucleotide
GSH: Glutathione
GSSG: Glutathione disulfide
FITC: Fluorescein isothiocyanate
BSD: Blasticidin S
MD: Molecular dynamics
RMSD: Root-mean-square deviation
DARTS: Drug affinity responsive target stability
Co-IP: Co-immunoprecipitation
IF: Immunofluorescence
TAI: Total abdominal irradiation
TBI: Total body irradiation
qRT-PCR: Quantitative real-time PCR
OA: Oleic acid
POA: Palmitoleic acid
SA: Stearic acid
UPS: Ubiquitin-proteasome system
SiRNA: small interfering RNA
SFA: Saturated fatty acids
PA: Palmitic acid
Rg: Radius of gyration
SCFA: Short-chain fatty acids
GUDCA: Glycoursodeoxycholic Acid
TUDCA: Tauroursodeoxycholic Acid
