# Supplementary material for: Gut Microbiota-Derived Tyrosol Alleviates Radiation-Induced Intestinal Injury via Targeting SCD1-MUFA Axis to Suppress ER Stress
Source: Int J Biol Sci. 2026 Feb 4;22(5):2469–91. doi: 10.7150/ijbs.126269 (PMC12965247; doi:10.7150/ijbs.126269)
Supplement: Supplementary file 1 — Supplementary figures and tables. [file ijbsv22p2469s1.pdf]

Supplementary tables and figures

Table S1. Primers for qRT-PCR

Name	Forward primer (5’-3’)	Reverse primer (5’-3’)
Actb	AGAAGCTGTGCTATGTTGCTCTA	AGACAGCACTGTGTTGGCATA
Il1b	TAGTCCTTCCTACCCCAATTTCC	TTGGTCCTTAGCCACTCCTTC
Il6	TAGTCCTTCCTACCCCAATTTCC	TTGGTCCTTAGCCACTCCTTC
Tnf	TTCTCATTCCTGCTTGTGGCA	ACTTGGTGGTTTGCTACGACG
Scd	TTCTTGCGATACACTCTGGTGC	CGGGATTGAATGTTCTTGTCGT
ACTB	CATGTACGTTGCTATCCAGGC	CTCCTTAATGTCACGCACGAT
SCD1	TTCCTACCTGCAAGTTCTACACC	CCGAGCTTTGTAAGAGCGGT

Table S2. Details of the web tools

Name	Version/Release	Accesse Date
PubChem	compound CID 10393 (tyrosol)	2024.03.14
ChemMapper	server snapshot Jan 2024	2024.03.18
HERB	2020-10 snapshot	2024.03.17
SEA (Similarity Ensemble Approach)	2023-12 release	2024.03.18
SwissTargetPrediction	2023 update	2024.03.19
SuperPred	v2.0	2024.03.19
GeneCards	v5.15	2024.03.16
Alliance of Genome Resources	rNov-2023	2024.03.16
DeepLoc-2.1	2023 release	2024.03.20

Table S3. Antibodies used in the experiments

Antibody	Supplier	Catalog	Application
SCD1	Abcam	ab236868	IB (1:1000), IF (1:200), IP (1:50)
β-actin	Abcam	ab6276	IB (1:1000)
β-tubulin	Abcam	ab7291	IB (1:1000)
DYKDDDDK tag	Proteintech	20543-1-AP	IB (1:1000)
VCP	Selleck	F1019	IB (1:1000)
PDI	Proteintech	66422-1-Ig	IF (1:200)
p-eIF2α	CellSignalingTechnology	3398	IB (1:1000)
eIF2α	CellSignalingTechnology	5324	IB (1:1000)
ATF4	CellSignalingTechnology	11815	IB (1:1000)
CHOP	CellSignalingTechnology	2895	IB (1:1000)
Vinculin	Proteintech	26520-1-AP	IB (1:1000)
ATF6	CellSignalingTechnology	65880	IB (1:1000)
IRE1α	CellSignalingTechnology	3294	IB (1:1000)
p-PERK	Proteintech	82534-1-RR	IB (1:1000)
PERK	CellSignalingTechnology	3192	IB (1:1000)

Table S4. Metabolites list in Fig. 1B heatmap

Number	Metabolites
1	C16 Sphinganine
2	PC(MonoMe(9,5)/MonoMe(9,5))
3	1b,3a,7b-Trihydroxy-5b-cholanoic acid
4	Alectinib
5	3a,4b,7a-Trihydroxy-5b-cholanoic acid
6	Tanacetin
7	Stercobilin
8	2b,3a,7a,12a-Tetrahydroxy-5b-cholanoic acid
9	Tomentolide A
10	2-Amino-N-[1-[[2-[[1-(2-hydroxyethylamino)-1-oxo-3-phenylpropan-2-yl]-methylamino]-2-oxoethyl]amino]-1-oxopropan-2-yl]-3-(4-hydroxyphenyl)propanamide
11	Fulvinervin B
12	Stercobilinogen
13	Terbucarb
14	Harderoporphyrinogen
15	Sycosterol A
16	Mesobilirubinogen
17	Betamethasone 17,21-dipropionate
18	3alpha,7alpha,11alpha-Trihydroxy-12-oxo-5beta-cholan-24-oic Acid
19	Nicansteroidin B
20	Trichostatin
21	N-(3-Hydroxypropyl)valine
22	Hydromorphone
23	2-[(4-{2-[(4-Cyclohexylbutyl)(cyclohexylcarbamoyl)amino]ethyl}phenyl)sulfanyl]-2-methylpropanoic acid
24	Americine
25	(3b,20R,22R)-3,20,27-Trihydroxy-1-oxowitha-5,24-dienolide 3-glucoside
26	(22E)-1alpha-hydroxy-24-oxo-26,27-cyclo-22,23-didehydrovitamin D3 / (22E)-1alpha-hydroxy-24-oxo-26,27-cyclo-22,23-didehydrocholecalciferol
27	3a,7b,21-Trihydroxy-5b-cholanoic acid
28	MG(20:4(5Z,8Z,11Z,14Z)/0:0/0:0)
29	Yamogenin
30	Erythro-5-hydroxy-L-lysinium(1+)
31	PC(0:0/PGE2)
32	8S-hydroxy-2E-Decene-4,6-diynoic acid
33	(2R,3Z)-Phycocyanobilin
34	Kanokoside D
35	Oxatomide
36	Lys-Gln-Ala-Gly-Asp-Val
37	1,3,7,12-Tetrahydroxycholan-24-oic acid
38	Unknown 370
39	Leucyl-Valine
40	Fukiic acid
41	(E)-C-HDMAPP
42	4-Bis(2-hydroxyethyl)amino-L-phenylalanine
43	Canarigenin 3-[glucosyl-(1->4)-6-deoxy-alloside]
44	Mycotoxin T 2
45	9Z,19Z-Heptatriacontadiene
46	n-iminoethyl-l-ornithine
47	Rockogenin
48	MGMG(16:2(7Z,10Z)/0:0)
49	7-O-Acetylaustroinulin
50	Taurolithocholic acid 3-sulfate

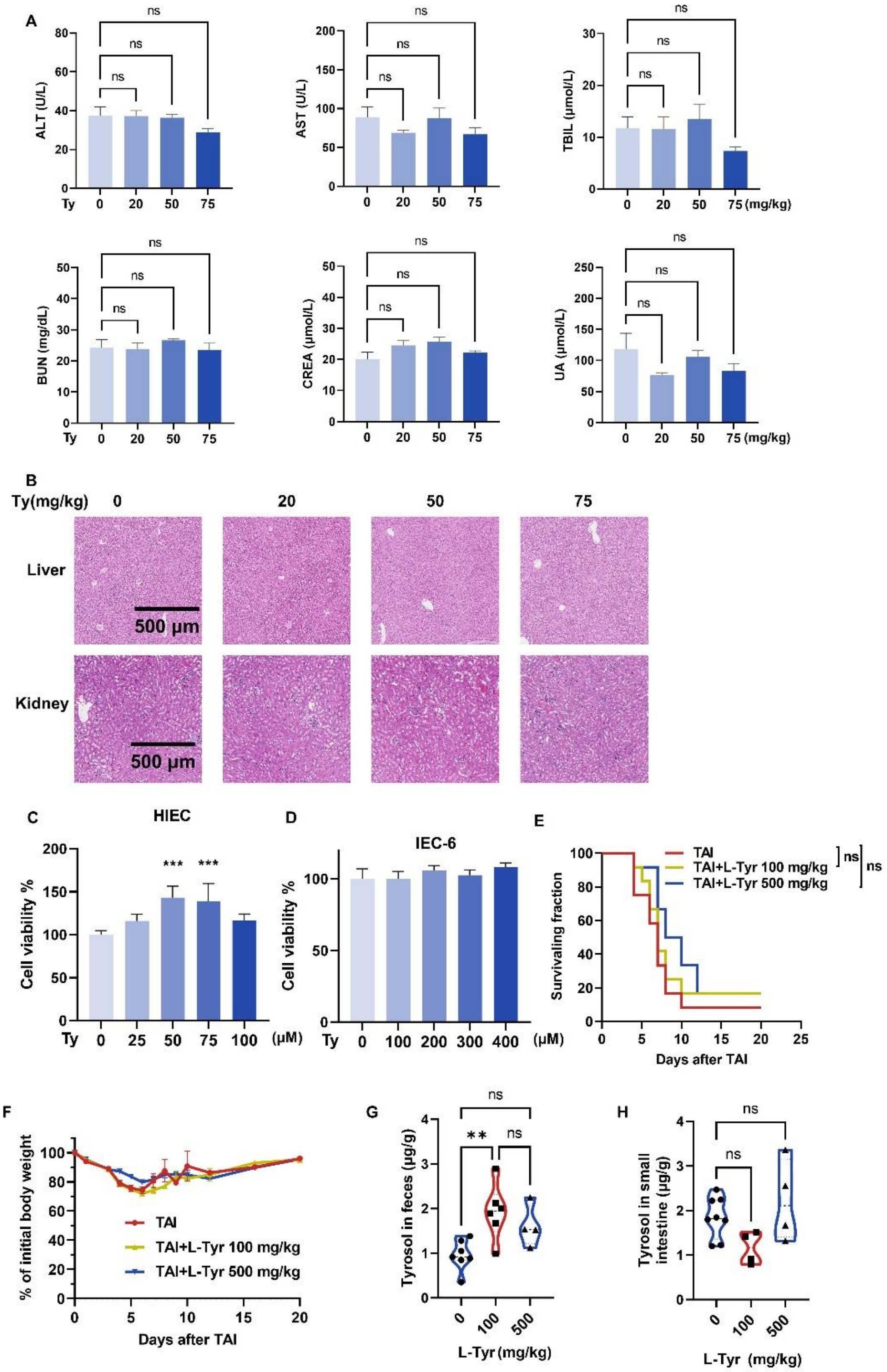

Figure S1. Effects of tyrosol gavage and L-Tyrosine intervention on mouse organ function and intestinal cell activity. (A) Serum levels of alanine aminotransferase (ALT), aspartate aminotransferase (AST), total bilirubin (TBIL), blood urea nitrogen (BUN), creatinine (CREA), and uric acid (UA) in mice at day 5 post tyrosol gavage (n=3 per group). (B) Representative H&E-stained liver and kidney tissues at day 5 post tyrosol gavage of mice (Scale bar = 500 μm). (C-D) CCK-8 assay of HIECs (C) and IEC-6 cells (D) treated with gradient tyrosol. (E) Survival curves of irradiated mice with/ without L-tyrosine treatment (n = 12 per group). (F) Body weight changes of irradiated mice. (G) Absolute quantification of tyrosol in feces from mice with/ without L-tyrosine treatment. (H) Absolute quantification of tyrosol in small intestine samples from mice with/without L-tyrosine treatment. Each symbol represents one mouse. Bars represent mean ± SD. Ty, tyrosol; L-Tyr, L-Tyrosine. ***$p < 0.001$, ns, no significant.

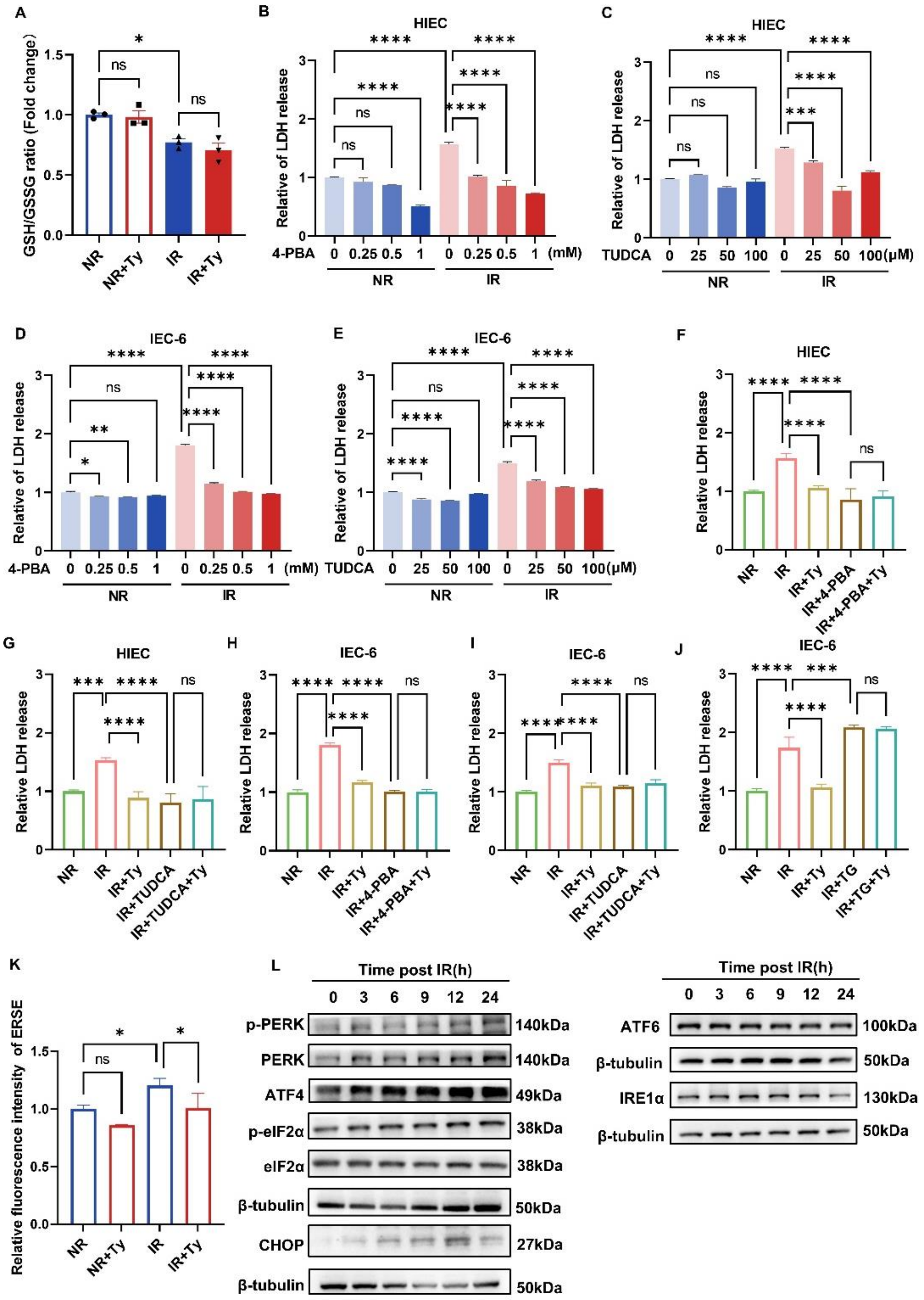

Figure S2. Tyrosol mitigates RIII by alleviate ER stress. (A) Glutathione (GSH) levels in the control or tyrosol (75 μM)-treated HIEC cells at 6 h after 0 or 8 Gy irradiation (IR). (B-E) LDH release assays of HIECs or IEC-6 cells pretreated with 4-PBA (B, D) or TUDCA (C, E), assessed at 72 h post-8 Gy IR. (F-I) LDH release assays of HIECs or IEC-6 cells pretreated with tyrosol combined with 4-PBA (F, H) or TUDCA (G, I), assessed at 72 h post-8 Gy IR. (J) LDH release assays of IEC-6 cells pretreated with tyrosol combined with TG, assessed at 72 h post-8 Gy IR. (K) Fluorescein reporter assay of tyrosol's effect on ER stress. HIECs were pretreated with tyrosol for 24h prior to IR. (L) Western blot assays of p-PERK, PERK, p-eIF2α, eIF2α, ATF4, CHOP, ATF6 and IRE1α in HIECs at 12 h post-IR. Bars represent mean ± SD. Ty, tyrosol; 4-PBA, 4-phenylbutyric acid; TUDCA, Tauroursodeoxycholate dihydrate**.** *$p < 0.05$, **$p < 0.01$, ***$p < 0.001$, ****$p < 0.0001$, ns, no significant.

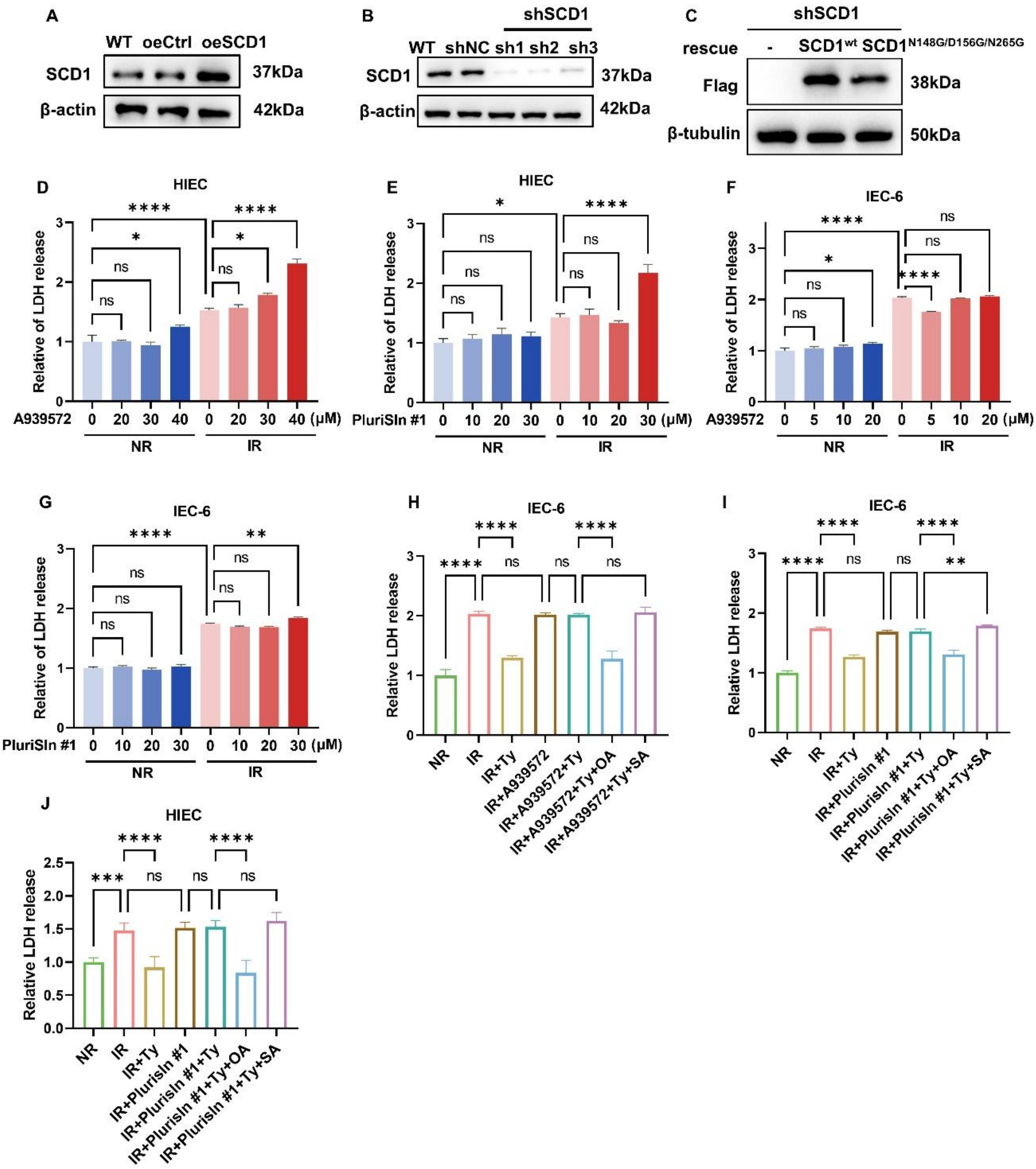

Figure S3. Tyrosol mitigates RIII in a SCD1-dependent manner. (A) Western blot assay of SCD1 levels in HIECs transfected with overexpression of SCD1 plasmid or control plasmids. (B) Western blot assay of SCD1 expression levels HIECs transfected with shRNA targeting SCD1 (shSCD1) or non-targeting controls (shNC). (C) Western blot assay of SCD1 levels in HIECs transfected with shSCD1 and rescued with $SCD1^{wt}$-Flag or $SCD1^{N148G/D156G/N265G}$-Flag overexpression. (D-G) LDH release assays of HIECs or IEC-6 cells pretreated with A939572 (D, F) or PluriSln #1 (E, G), assessed at 72 h post-8 Gy IR. (H-J) LDH release assays of IEC-6 (H, I) cells or HIECs (J) pretreated with tyrosol combined with A939572, PluriSln #1, OA, or SA, detected at 72 h post-IR. Bars represent mean ± SD. Ty, tyrosol; OA, oleic acid; SA, stearic acid**.** *$p < 0.05$, **$p < 0.01$, ***$p < 0.001$, ****$p < 0.0001$, ns, no significant.

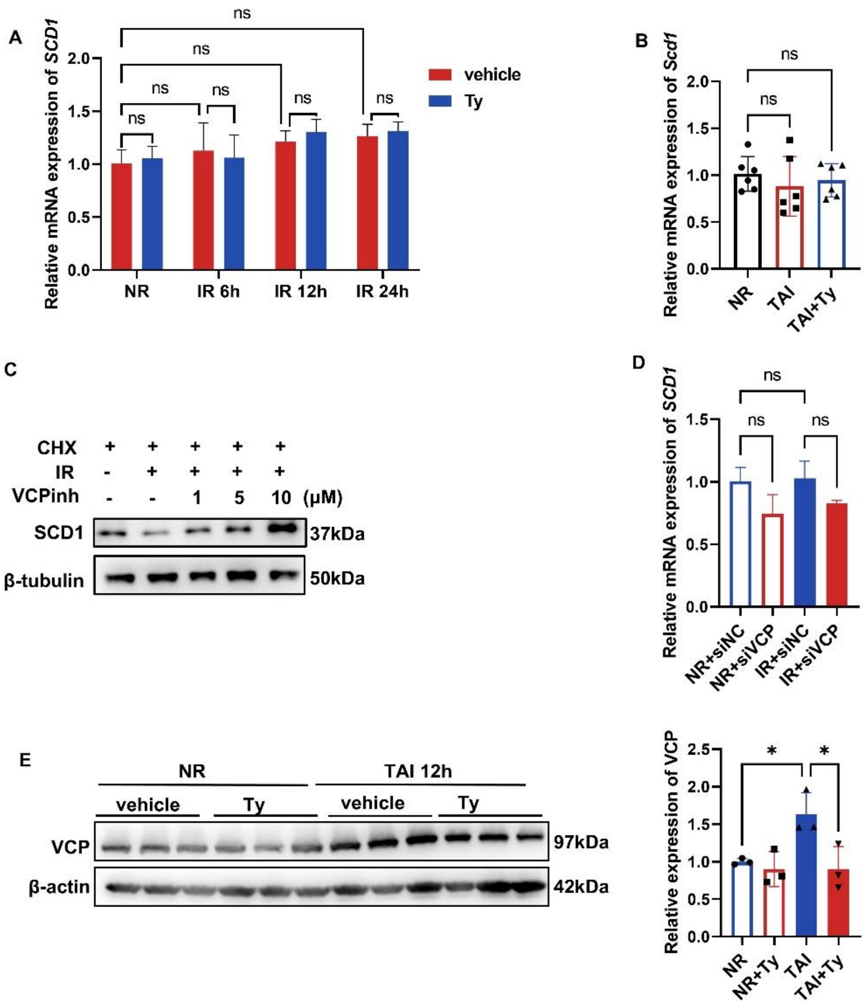

Figure S4. Tyrosol inhibits VCP-mediated degradation of SCD1. (A) qRT-PCR analysis of SCD1 mRNA in HIECs following tyrosol treatment and irradiation (IR). (B) qRT-PCR analysis of *SCD1* mRNA expression in mouse small intestines under identical conditions. (C) Western blot assay of SCD1 in HIECs at 12 h post-IR (8 Gy), following treatment with VCP inhibitor NMS-873 (10 μM, 2 h). (D) qRT-PCR analysis of *SCD1* mRNA expression in HIECs transfected with siRNA against VCP or non-targeting controls for 72 h. (E) Western blot assay of VCP in mouse small intestines at 12 h post-12 Gy TAI. Left: representative images; right: VCP levels normalized to β-actin relative to controls (n=3 per group). Bars represent mean ± SD. Ty, tyrosol; CHX, cycloheximide; VCPinh, VCP inhibitor. *$p < 0.05$, ****$p < 0.0001$, ns, no significant.
